# Water Nanoconfined in a Hydrophobic Pore: Molecular Dynamics Simulations of Transmembrane Protein 175 and the Influence of Water Models

**DOI:** 10.1021/acsnano.1c06443

**Published:** 2021-11-16

**Authors:** Charlotte I. Lynch, Gianni Klesse, Shanlin Rao, Stephen J. Tucker, Mark S. P. Sansom

**Affiliations:** 1 Department of Biochemistry, University of Oxford, South Parks Road, Oxford, UK, OX1 3QU; 2 Clarendon Laboratory, Department of Physics, University of Oxford, Parks Road, Oxford, UK, OX1 3PU

**Keywords:** hydrophobic gating, water, nanoconfinement, polarizability, ion channel, TMEM175

## Abstract

Water molecules within biological ion channels are in a nano-confined environment and therefore exhibit behaviours which differ from that of bulk water. Here, we investigate the phenomenon of hydrophobic gating, the process by which a nanopore may spontaneously de-wet to form a ‘vapour lock’ if the pore is sufficiently hydrophobic and/or narrow. This occurs without steric occlusion of the pore. Using molecular dynamics simulations with both rigid fixed-charge and polarizable (AMOEBA) force fields, we investigate this wetting/de-wetting behaviour in the TMEM175 ion channel. We examine how a range of rigid fixed-charge and polarizable water models affect wetting/de-wetting in both the wild-type structure and in mutants chosen to cover a range of nanopore radii and pore-lining hydrophobicities. Crucially, we find that the rigid fixed-charge water models lead to similar wetting/de-wetting behaviours, but that the polarizable water model resulted in an increased wettability of the hydrophobic gating region of the pore. This has significant implications for molecular simulations of nano-confined water, as it implies that polarizability may need to be included if we are to gain detailed mechanistic insights into wetting/de-wetting processes. These findings are of importance for the design of functionalised biomimetic nanopores (for *e*.*g*. sensing or desalination), as well as for furthering our understanding of the mechanistic processes underlying biological ion channel function.

## Introduction

**Figure 1:**
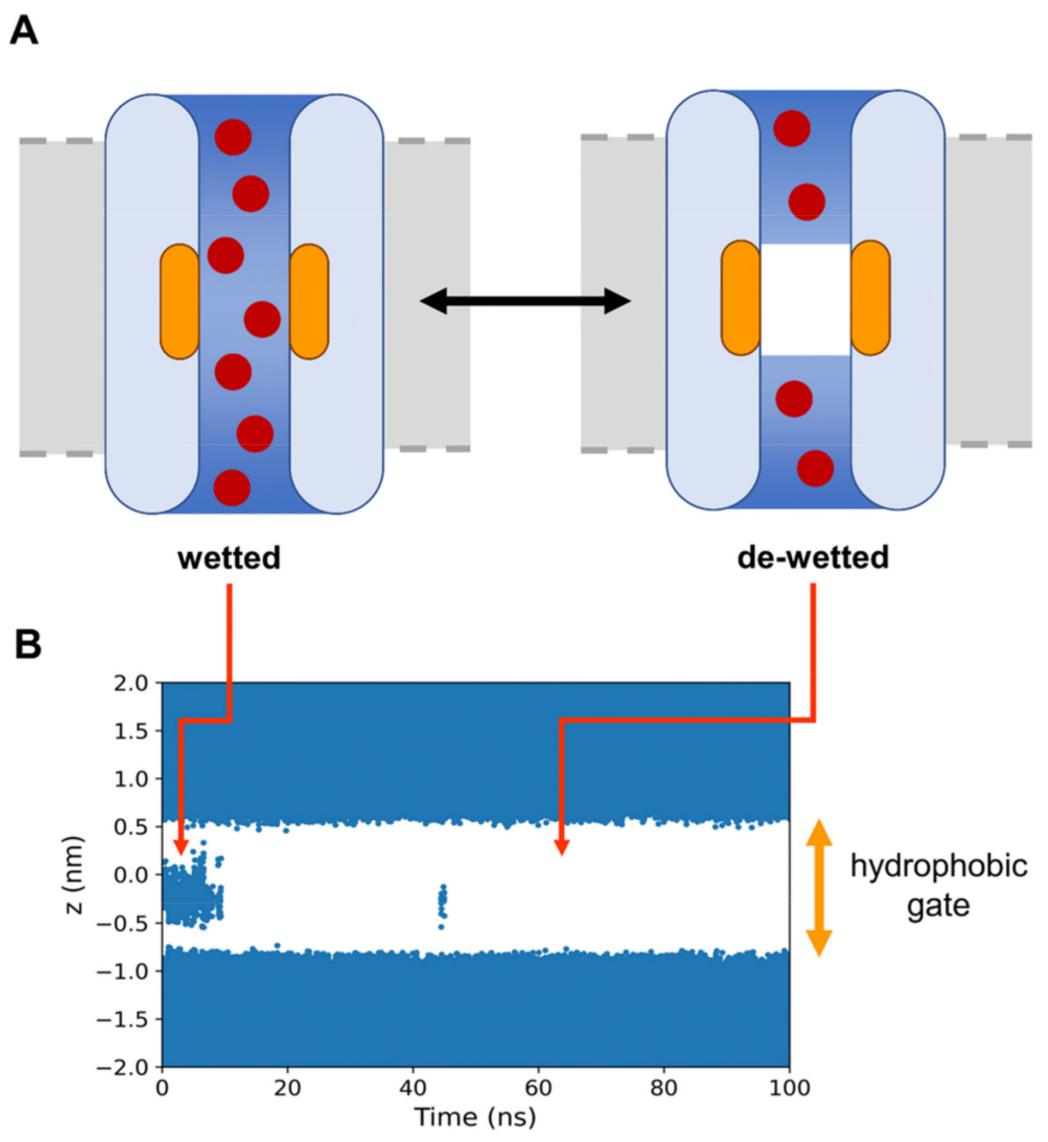
**A** The principle of hydrophobic gating: in the wetted state, both water (medium blue) and ions (red) can pass through the ion channel (pale blue) and the hydrophobic gate (orange); whereas in the de-wetted state, the channel, though not physically occluded, is closed to the passage of ions. **B** An example of simulated de-wetting of a hydrophobic gate. The trajectories of water molecules (dark blue) around and initially located in a hydrophobic gating region (indicated by the orange arrow, centred at *z* = 0) are shown projected onto the *z* axis. The data are taken from a simulation of the TMEM175 channel protein using the TIP4P water model (see main text for details; also see SI Fig. S1).

The transport behaviour of water and other liquids in nanoscale pores is of both fundamental and technological importance.^1^ Nanoscale pores across membranes are of especial interest.^2^ In biological systems, channels are protein nanopores which enable the flow of water molecules and/or ions across cell membranes.^3^ The behaviour of water molecules within nanoscale pores differs in several respects from that of bulk water.^4–7^ In particular, ion channel proteins and nanopores may possess a hydrophobic gate (Fig. 1A). This is a constricted region of the pore which is lined with hydrophobic residues in which de-wetting may occur to form a “vapour lock”.^8–9^ This in turn presents an energetic barrier to ion permeation and thereby functionally closes the channel. Therefore, in these ion channels, the hydrophobic gate closes the pore to the passage of water and ions without the need for steric occlusion of the pore.^10^ The presence of hydrophobic gates in several ion channel species has been inferred from both computational and experimental studies. Hydrophobic gating has also been designed into synthetic nanopores *e*.*g*. ^11^. Thus, a detailed mechanistic understanding of hydrophobic gating is relevant both to studies of ion channel structure/function relationships,^12^ and also to the design of gating functionality^13^ into synthetic nanopores.^14–15^

The behaviour of a hydrophobic gate has been shown to depend critically on the radius of the pore and on the hydrophobicity of the pore lining.^16–22^ Furthermore, in addition to these two structural parameters, the application of an electric field^23–24^ or pressure^25–26^ can cause the pore to wet, thereby opening an otherwise closed channel to the passage of water and ions.

Molecular dynamics (MD) simulations have been widely applied to explore the parameters affecting hydrophobic gating and the wetting/de-wetting behaviour both of ion channels and of simplified models of nanopores (see *e*.*g*. ^6, 12^ for two recent reviews). By measuring the number density of water molecules within the gating region, an associated energetic barrier to wetting can be estimated.^27^ This has been used to characterise the likely functional state of nearly 200 experimentally determined ion channel structures,^22^ leading to a more global analysis of the dependence of hydrophobic gating behaviour (in terms of the energetic barrier to wetting) on channel structure. MD simulations have also been used to explore the effect of pore lining mutations on hydrophobic gating in *e*.*g*. the bestrophin-1 chloride channel.^28^

When conducting MD simulations of water behaviour in nanopores and channels, a crucial but not yet widely investigated consideration is the choice of water model. For example, recent simulations have examined the effect of varying water models on the behaviour of water and ions within cyclic peptide nanopores^29^ and within ligand gated ion channels.^30^

Due to the competing effects of hydrogen bonding, van der Waals and other intermolecular interactions, water is a difficult molecule to model accurately. To date, there are over 130 different water models for use in MD simulations and related calculations, each with a range of physical properties which are comparable to experimental values.^6^ Typically, these water models are developed to accurately reproduce one or more experimental properties of bulk water. In the case of water in hydrophobic gates, however, water molecules are confined within a region of ~1 nm diameter and ~2 nm in length. This means that there are likely to be only a small number of water molecules in the hydrophobic gating region, with a maximum of ~50 for a cylinder of this volume. Water confined within such a nanoscale channel is unlikely to behave as it does in bulk, *e*.*g*. in terms of its dielectric behaviour.^7^ Consequently, it is not certain that water models developed to reproduce bulk behaviour will be directly transferable to such nanoconfined environments. Initial studies on the pore domain of the 5-HT_3_ (5-hydroxytryptamine) receptor suggest that the choice of water model can have an effect on the wetting/de-wetting behaviour, particularly in the case of structures which are in an intermediate open/closed state.^30^ (It should be noted that the 5-HT_3_ receptor system corresponds to a modest degree of nanoconfinement, with ~17 water molecules in the key hydrophobic region of the pore). In particular, there are indications that the use of more sophisticated water models such as polarizable water models (the dipole moment of which will be responsive to changes in local environment) may be required to capture more adequately the interactions within hydrophobic gates. Although polarizable models are more computationally expensive, the development of more powerful computing facilities and of improved software enables the use of such models for complex biological systems.^31–32^ Thus, recent studies have explored the influence of polarizable models on *e*.*g*. simulations of the interactions of ions with lipid bilayer membranes,^33^ simple model ion channels *e*.*g*. ^34^ and on electric field strength within the active site of an enzyme.^35^ The effect of polarizable water models within synthetic nanoconfined environments under an electric field has also been investigated, with the conclusion that polarizability can affect the structure and dynamics of the systems.^36–38^ Various rigid fixed-charge water models have also been assessed for ligand binding in host/guest systems^39^ and for water behaviour in peptide nanotubes.^29^

Wetting/de-wetting of the well-defined hydrophobic gate in the TMEM175 (transmembrane protein 175) ion channel^28, 40–43^ provides an extreme case of non-bulk behaviour of water when nanoconfined in a pore. In this structure the hydrophobic gate is particularly small, with only 1-4 waters able to be accommodated at any one time. We have therefore used this hydrophobic gate to assess how different rigid fixed-charge water models perform compared to the AMOEBA^44–45^ polarizable water model in a highly nanoconfined environment.

In the current study we use the prokaryotic CmTMEM175 structure.^40^ We investigate the effects of changing both the pore-lining residues and the water model employed in the simulations on wetting/de-wetting behaviour of this hydrophobic gate. This region of the channel is formed by three hydrophobic rings (each with four-fold symmetry): one ring of isoleucine residues (residue Ile23) followed by two of leucine residues (Leu27 and Leu30 respectively). We explore how mutating this hydrophobic gate to either three rings of valines (slightly smaller hydrophobic sidechains), or alanines (hydrophobic but much smaller sidechains), or asparagines (hydrophilic sidechains) affects the water behaviour. We also investigate the transferability of different rigid fixed-charge (*i.e*. additive) and polarizable water models for simulating water behaviour within the hydrophobic gate, *i.e*. we evaluate whether the different water models produce consistent wetting/de-wetting behaviours. Our results provide mechanistic insights into the behaviour of water within a highly nanoconfined pore. This is of direct relevance to the ongoing interest in nanoconfined water as studied either experimentally (*e*.*g*. ^46–47^) or computationally (*e*.*g*. ^7, 48^).

## Results and Discussion

### Channel System and Simulations

**Figure 2:**
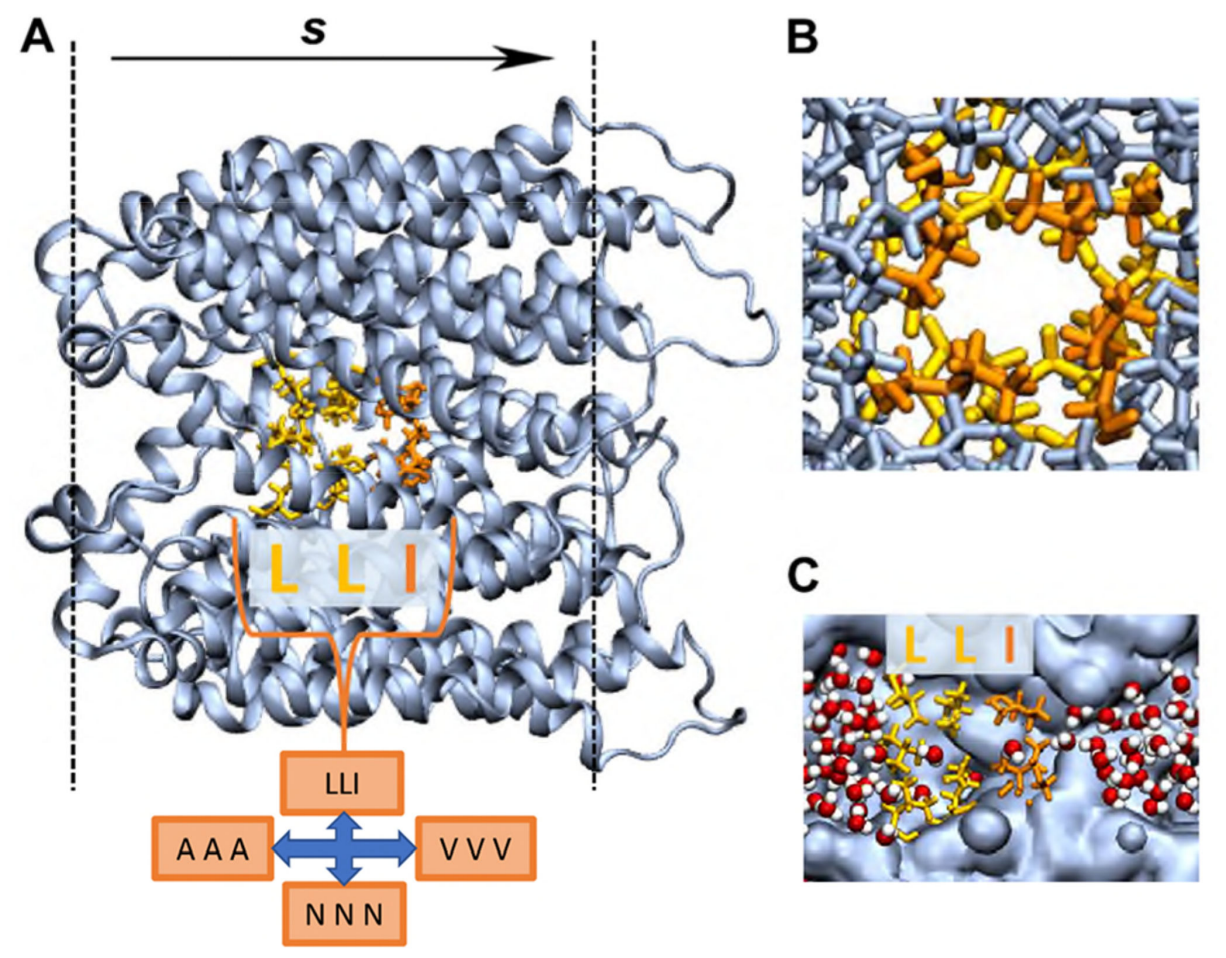
**A** The simulation set-up with the TMEM175 protein in blue and the wild-type hydrophobic gating residues highlighted in yellow (LEU) and orange (ILE). The LEU and ILE residues in the hydrophobic gate (LLI) are substituted for ALA, ASN and VAL respectively to create three mutants (AAA, NNN and VVV respectively). The position of the lipid headgroups in the bilayer are denoted by the dashed black line, and *s* indicates the path direction through the centre of the pore. **B** A close-up looking down the wild-type pore. **C** A slice through the pore with water (red spheres for oxygen, white spheres for hydrogen) exiting the hydrophobic gating region of the pore surface (blue).

In this study we used the prokaryotic CmTMEM175 (PBD: 5VRE; 3.3 Å resolution)^40^ as a model for a pore with a hydrophobic gate which can be wetted/de-wetted.^28^ In this channel structure the first transmembrane helix from each of four subunits line a central pore with a constriction (with a minimum radius of < 0.15 nm on the crystal structure) formed by three rings of hydrophobic sidechains: Ile23, Leu27 and Leu30 (see Fig. 2). In two other TMEM175 channels these rings of residues are either conserved (in hTMEM175, PDB: 6WC9) or replaced by three rings of leucines (in MtTMEM175, PDB: 6HD8). However, the structural conservation of this region is less clear-cut,^40–42^ as might be expected for a hydrophobic gating region.

In order to explore systematically some of the parameters affecting hydrophobic gating (*e*.*g*. pore radius and hydrophobicity), the CmTMEM175 structure (PDB code 5VRE) was embedded within a phosphatidylcholine (PC) bilayer and solvated with a ~0.15 M NaCl aqueous solution. In these simulations, backbone restraints were applied to maintain the experimentally observed polypeptide backbone conformation whilst allowing a degree of sidechain flexibility/mobility. In initial simulations of the wild type (*i.e*. native) protein the gating region was seen to be sufficiently narrow and hydrophobic that the pore spontaneously de-wetted early on in simulations (Fig. 1B and SI Fig. S1A).

As can be seen (Fig. 2), the hydrophobic gate in the CmTMEM175 structure is formed by a constricted region of three rings of hydrophobic sidechains, forming an ‘ILL’ motif. In order to explore the sensitivity of wetting/de-wetting behaviour to the nature of this motif, these residues were *in silico* mutated to either three rings of valines (VVV), *i.e*. hydrophobic sidechains but smaller than isoleucine or leucine, or three rings of alanines (still hydrophobic, but substantially smaller sidechains), or three asparagines (the sidechain of which is the same size as that of leucine, but is polar and able to form H-bonds): see Fig. 2A. For both the wild type and the three mutant channels we conducted simulations with various water models (see below). Simulations were of 100 ns duration with 6 repeats for fixed-charge water models and of up to 50 ns duration with 3 repeats for the polarizable AMOEBA model.

### Effect of Mutations on Wetting/De-wetting

**Figure 3:**
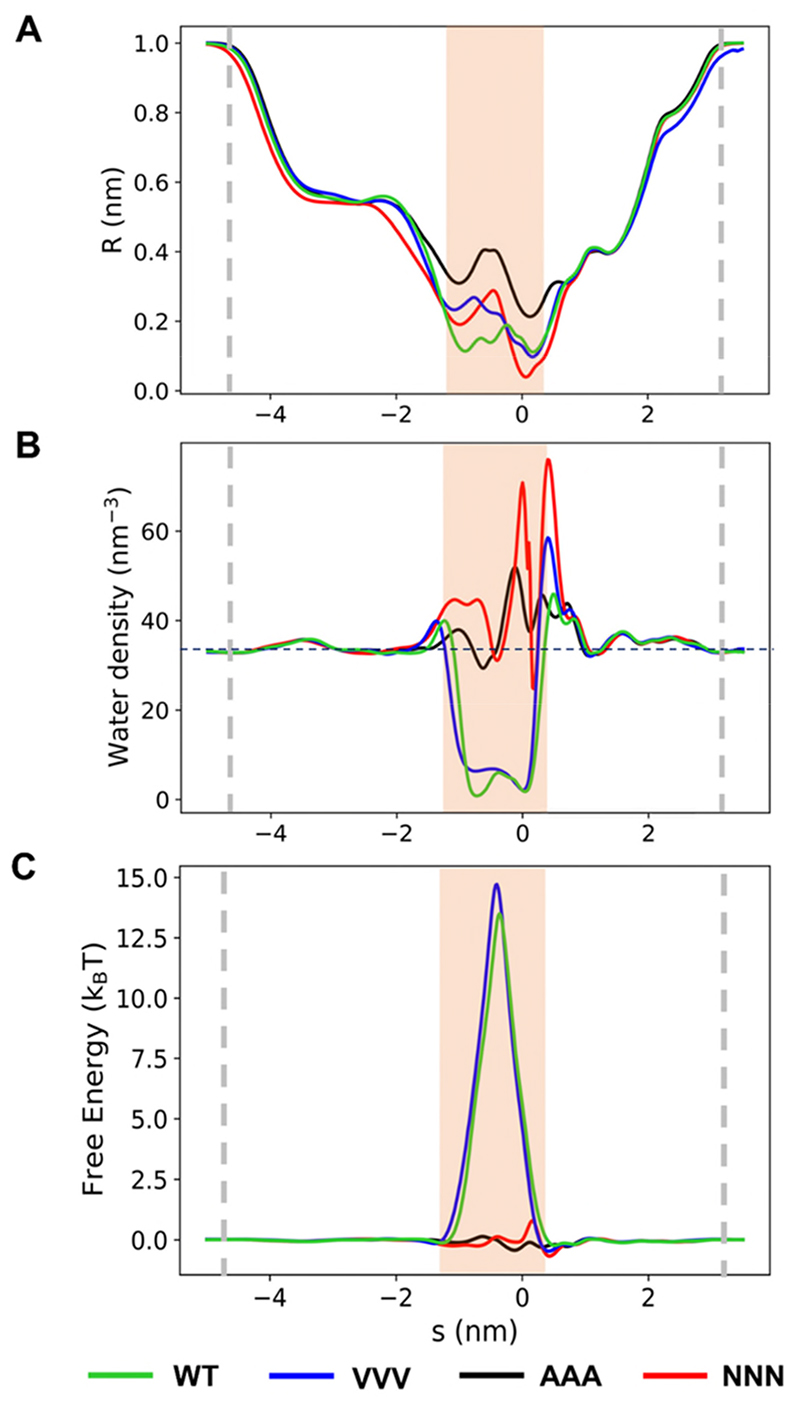
Profiles of pore radius (**A**), water density (**B**) and water free energy (**C**) as a function of distance along the pore centre line, s. The profiles for the wild-type structure (WT, green) and AAA (black), NNN (red) and VVV (blue) mutants are shown, each averaged over the last 90 ns of the simulation and subsequently averaged over 6 independent repeats. The water model TIP4P/2005 was used for all simulations. The hydrophobic gating region is indicated by the orange band, and the approximate positions of the lipid head groups are denoted by the grey dashed lines.

In simulations of the wild-type channel, the hydrophobic gate is associated with a constriction of radius down to 0.1-0.2 nm (Fig. 3A). This is comparable to the radius of a water molecule (0.14 nm). The sidechains of the isoleucines and leucines result in a highly hydrophobic lining. Thus, in simulations using the TIP4P/2005 water model and OPLS all-atom protein force field (see Methods below), the gating region de-wets within the first ~10 ns for most of the repeats (SI Fig. S1B). The gating region remains relatively dry for the remainder of each of the simulations. However, in some repeats of the simulations, a water molecule is temporarily trapped within the gate, due to the tight constrictions of ~0.1 nm at each end of the gate. Consequently, the time averaged water density (Fig. 3B) does not completely fall to 0 nm^-3^ within the hydrophobic gate of the wild-type channel (this will be discussed in more detail below). The energetic barrier to water of this region (Fig. 3C) is 13.5 (± 4.8) k_B_T (averaged over the 6 repeats, ± standard deviation). This conformation of the pore therefore clearly corresponds to a functionally closed state.

The constriction of the pore in the ‘VVV’ mutant remained hydrophobic but was a little wider (by ~0.1 nm at the lower/extracellular (*i.e. s* = -1.3 nm) end of the gate (Fig. 3A). Despite this small increase in radius, the pore remained de-wetted (Fig. 3B), with an energetic barrier comparable to that of the wild-type channel (Fig. 3C).

In contrast, although the AAA mutant preserved the hydrophobic nature of the pore lining, it was wider, with two minima (Fig. 3A) of ~0.2 and ~0.3 nm radius. This was sufficient for the pore to remain fully wetted (Fig. 3B), thus presenting no barrier to ion permeation (Fig. 3C). The more polar/hydrophilic mutant, NNN, had a pore radius profile comparable to that of the VVV mutant (Fig. 3A). However, the hydrophilic lining provided by the Asn (NNN) sidechains (to which water can form H-bonds) was such that the pore remained fully wetted (Fig. 3B) with no energetic barrier to water permeation (Fig. 3C).

### Effect of Rigid Fixed-charge Water Model on Wetting/De-wetting in the Wild-type Channel

**Figure 4:**
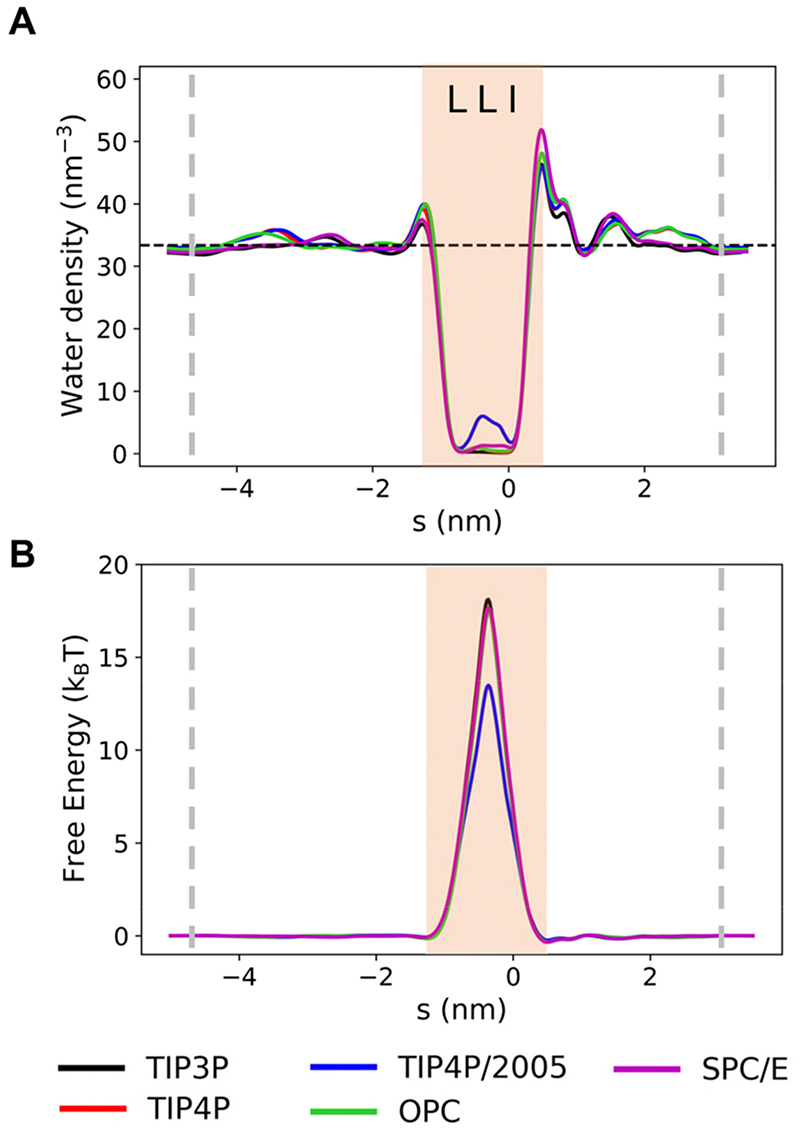
The effect of changing the rigid fixed-charge water model on the water density (**A**) and free energy (**B**) profiles for the wild-type TMEM175 structure. The position of the hydrophobic gate is shaded in pale orange, and the approximate position of the lipid head groups is given by the grey dashed lines. (See also SI Figs. S2, S3 and S4).

The results discussed so far employed the rigid fixed-charge (*i.e*. additive) TIP4P/2005 water model, which has been shown to perform well over a range of (bulk) water properties^49–50^ and has been used in recent studies of nanoconfined water *e*.*g*. ^51^. To explore the robustness of these results to variations in rigid fixed-charge water models, we investigated the effect of five different models on the de-wetting behaviour in the hydrophobic gate of the wild-type CmTMEM175 structure (Fig. 4). These models were chosen to cover a broad range of model types (see *e*.*g*. ^6, 52^ for useful summaries). TIP3P and SPC/E are well-established 3-point water models with differing geometries: TIP3P^53^ is based on gaseous water, while SPC/E^54–55^ is based on ice. TIP4P and TIP4P/2005 are 4-point water models, also based on the geometry of TIP3P but with an extra particle to displace the partial charge associated with the oxygen away from the oxygen site. TIP4P, along with TIP3P, is routinely used in biomolecular simulations, whereas the more recently developed TIP4P/2005 model is not yet commonly used. OPC is a recently developed 4-point model with an unusual geometry.^52, 56–57^ The latter includes a bond angle of 103.6°, smaller than the gaseous bond angle of 104.5°, and a smaller than expected O-H bond length of 0.8724 Å. The OPC geometry was developed by optimising the point charge arrangement in comparison to quantum mechanical calculations.^52^

For all five rigid fixed-charge models, the pore de-wetted as evidenced by the water density profiles (Fig. 4A) and the associated free energy barriers (Fig. 4B). In the case of TIP4P/2005, some water molecules were trapped by the constrictions at the ends of the hydrophobic gate, as discussed above, resulting in a decrease in the free energy barrier. This difference is however just within the limit of the standard deviations (averaged over repeats) for the five water models (see SI Figs. S2, S3 and S4, and therefore we attribute this to stochastic variation rather than a significant difference due to the water model. We therefore conclude that the wetting/de-wetting behaviour is not substantially affected by changes in additive water model, and that these water models are equally valid for investigating the effects of hydrophobic gating. In the absence of direct experimental data, it is difficult to assess whether the water models used are transferrable from bulk to nano-confined (*i.e*. channel) systems. However, recent experimental studies have attempted to estimate water profiles within the hydrophobic gate of an ion channel^58^ (but not yet for TMEM175). In the meantime, we can state that the five additive water models explored appear to be equally transferrable when moving from bulk water to water confined in a nanopore.

**Figure 5:**
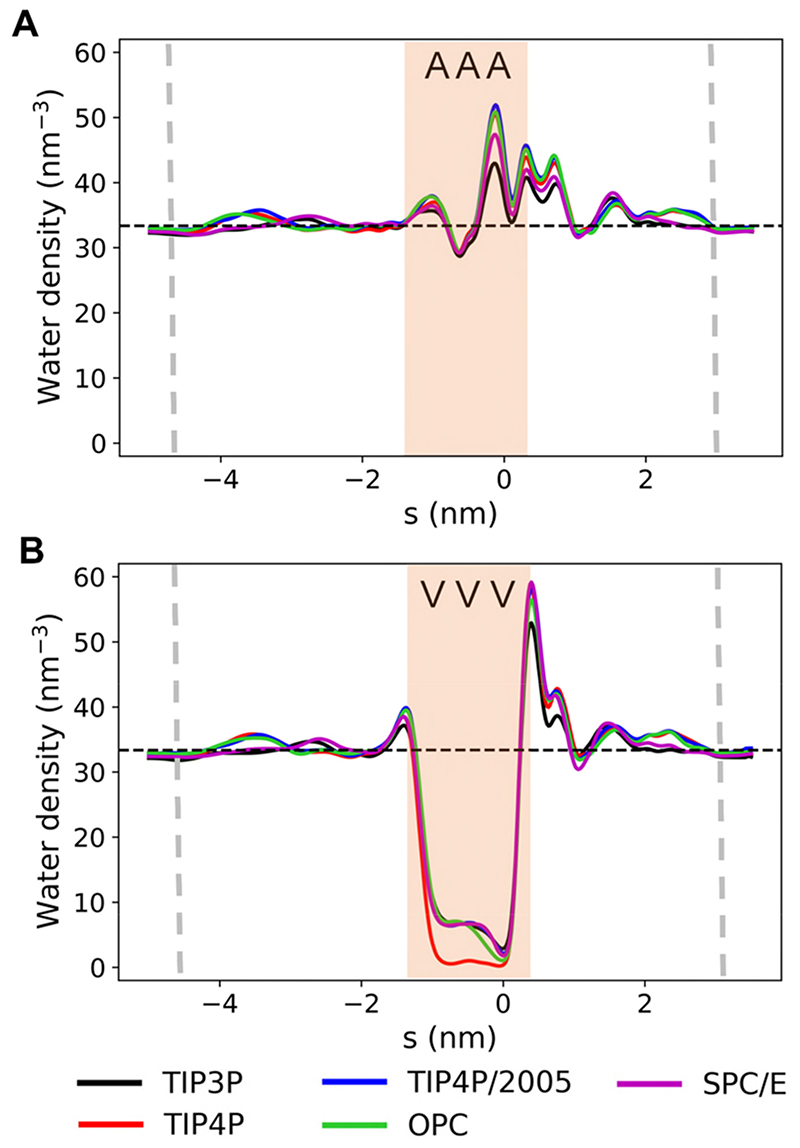
Water density profiles for different rigid fixed-charge water models for the AAA mutant (**A**) and the VVV mutant (**B**). Bulk water density is denoted by the black dashed line. The position of the hydrophobic gate is shaded in pale orange, and the approximate position of the lipid head groups is given by the grey dashed lines. (See also SI Figs. S5-10.)

Comparing the five rigid fixed-charge water models in the mutants also produced consistent results. For example, in the case of the AAA (Fig. 5A and SI Fig. S5 & S6) and NNN (SI Fig. S7 & S8) mutants, the hydrophobic gate remains wetted across the five water models. Similarly, in the VVV mutant, the hydrophobic gate de-wets in all cases (Fig. 5B and SI Fig. S9 & S10). Any differences between water model across the free energy profiles are well within the standard deviations and are therefore due to stochastic variation rather than due to the choice of water model (see SI Figs. S6, S8 & S10).

### Effect of a Polarizable Water Model on Wetting/De-wetting

We are especially interested in how polarizable water would behave in the TMEM175 hydrophobic gate structure given the stochastic observation of a ‘trapped’ water (or waters) in this extreme example of nanoconfinement, as seen in our simulations using *e*.*g*. the TIP4P/2005 water model (see above and SI Fig. S1B). Experimentally, water molecules in hydrophobic nanoslits exhibit an anomalously low dielectric constant largely reflecting their rotational immobilisation.^46^ Simulation studies have been used to compare a rigid fixed-charge (SPC/E) water model and a flexible polarizable (ABEEM-7P) model for water molecules confined in graphene nanocapillaries with a width ranging from 0.59 to 1.1 nm. In the latter case the polarizable model resulted in more ordered water than with SPC/E.^59^

**Figure 6:**
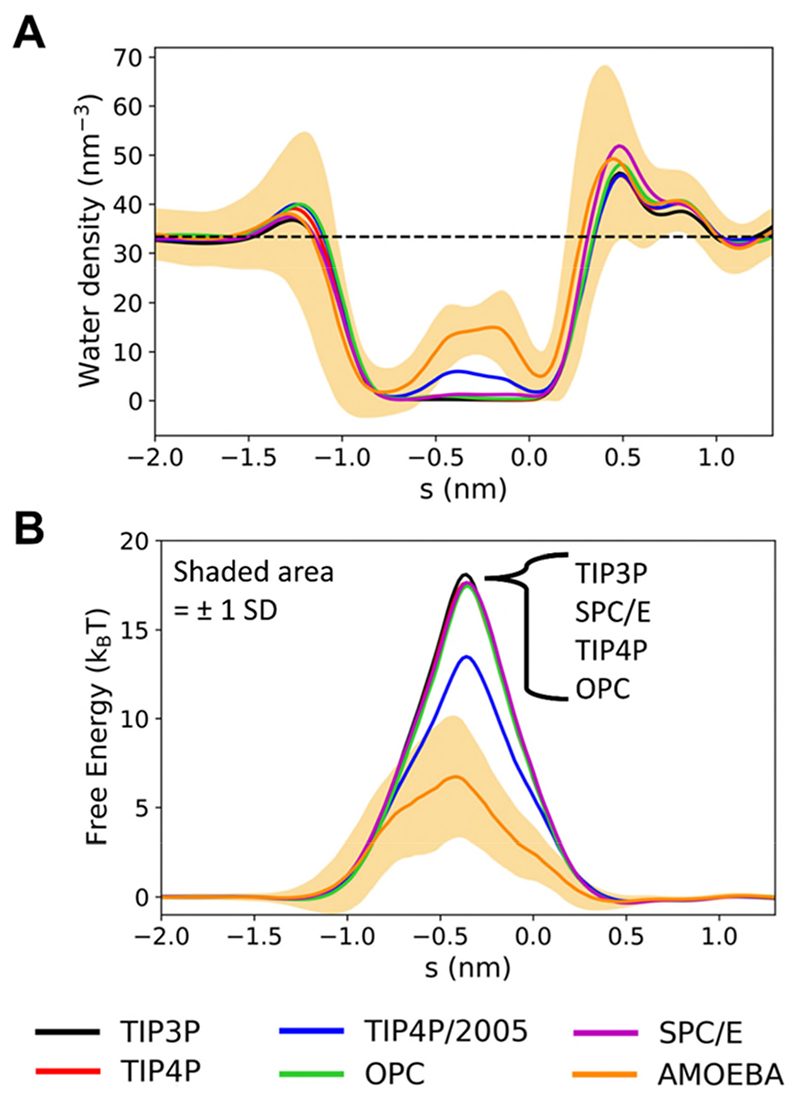
Comparison of water density (**A**) and free energy (**B**) profiles for the TIP4P/2005 fixed-charge and the AMOEBA polarizable water models for the wild-type TMEM175 structure. The shaded regions denote ±1 standard deviation calculated over all simulation frames included in the analysis (discarding the first 10 ns as equilibration), and averaged over all repeats. The dashed black line denotes the value of bulk water density.

**Figure 7:**
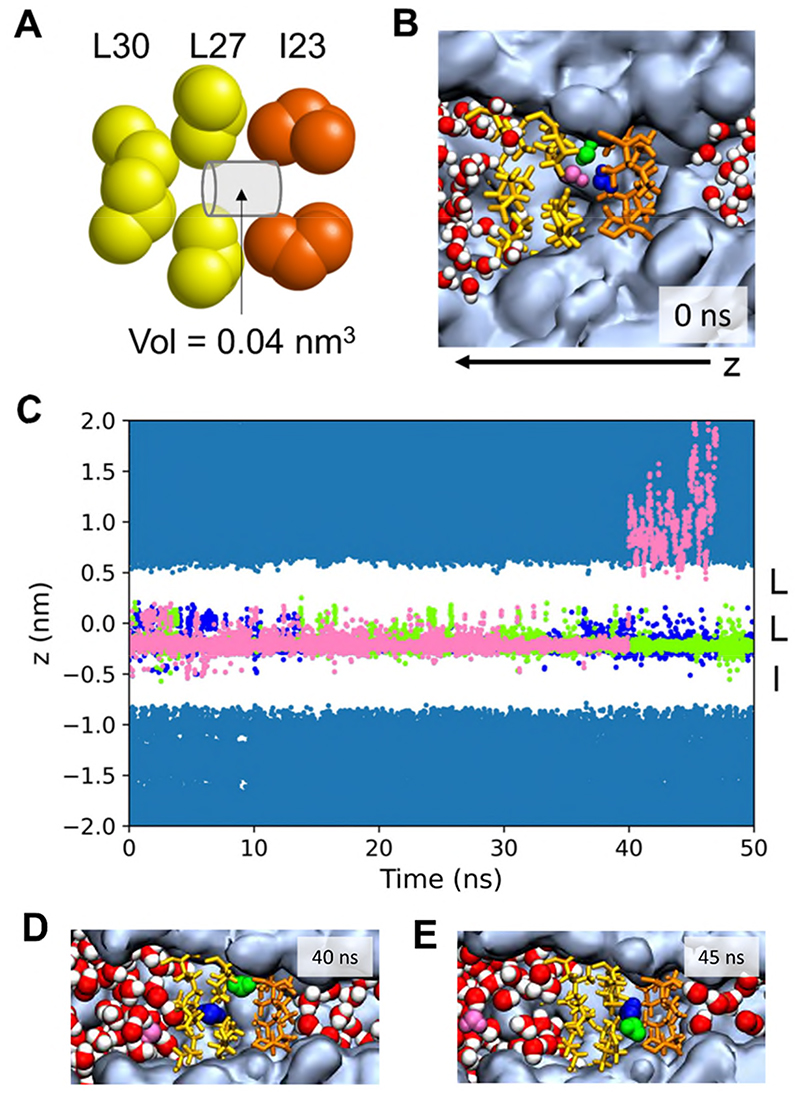
**A** Schematic of the ‘extreme nanocavity’ in the TMEM175 hydrophobic gate, showing the cylindrical approximation to the WT cavity volume. **B** Snapshot of three ‘trapped’ water molecules (coloured blue, pink and green) within the nanocavity which partially hydrate the hydrophobic gate in the AMOEBA simulations. **C** Trajectories of the three trapped waters (coloured as in **B**) for an AMOEBA simulation. The pink water molecule can be seen to leave after ~40 ns. **D and E** Snapshots of the simulation in **C** immediately after and ~5 ns after the pink water molecule has departed from the nanocavity, showing the relaxation of the two remaining water molecules so that they sit between the I32 and L27 sidechain rings. (See also SI Fig. S11).

Our simulations of the extreme nanoconfinement within the TMEM175 gate (the radius in the middle of the hydrophobic gate is ~0.16 nm for the wild-type (WT) channel, comparable to that of a single water molecule) reveal a difference in the de-wetting behaviour when comparing the polarizable AMOEBA14 water model^45^ to the five additive water models. This is most clear cut for the WT channel, for which the pore radius profiles within the hydrophobic gate are identical for the five non-polarizable and for the polarizable (AMOEBA) models (SI Fig. S2A). For this channel, the number density of water in the nanocavity in the centre of the hydrophobic gate (Fig. 6A) was ~0 nm^-3^ for TIP4P, ~6 nm^-3^ for TIP4P/2005 and ~15 nm^-3^ for AMOEBA, corresponding to free energies along *s* of ~18 (±4), ~13 (±5) and ~7 (±4) kBT respectively, where the numbers in parentheses are ± 1 standard deviation averaged over repeat simulations (Fig. 6B). Thus, water within this extreme nanoconfinement (Fig. 7A; the volume of the nanocavity is ~0.04 nm^3^, *i.e*. just above the volume occupied by a single water molecule in the bulk state, 0.03 nm^3^) is stabilized by on average 6 to 11 kBT (*i.e*. 15 to 28 kJ/mol) by the use of a polarizable model. Of course, this is an approximate estimate, assuming three to six repeat simulations provide adequate sampling. FEP calculations could, for example, be used to estimate this more exactly.

For the VVV simulations (SI Fig. S9 and S10) the situation was less clear. With this (modelled) mutant pore there was a degree of dependence of the pore radius profile on the water model employed which complicated such detailed comparison.

Overall, this suggests that the use of a polarizable water model has a significant effect on the de-wetting/wetting behaviour of a ‘tight’ hydrophobic gate, and that future simulations of hydrophobic gating in general would benefit from a systematic investigation into the effects of including molecular polarizability. It is also worth noting that, whilst the protein/lipid/ion force field was consistent for the rigid fixed-charge water models (OPLS with united-atom lipids), in the case of our polarizable simulations *all* atoms were modelled with the AMOEBA force field (see Methods). Therefore, it cannot be ruled out that the induced polarizability of the protein also affected the wetting/de-wetting behaviour. This could be assessed by running simulations using a combination of polarizable and non-polarizable force fields within a simulation. Daub *et al*. have investigated the combination of a polarizable water model with non-polarizable ions in a silica nanopore, and their results suggest that the polarizability of the water has a greater effect over that of the ions.^36^

We can make partial comparisons with other studies. Previous simulation studies of the effect of including polarizability focussed on a less extreme case of nanoconfinement^30^ involving the gating region of a M25 nanopore derived from the (partially) open state of the 5HT_3_R channel (PDB 6DG8). For this channel system the time-averaged water density was respectively ~22 nm^-3^ (for TIP3P water), ~16 nm^-3^ (TIP4P/2005) and ~35 nm^-3^ (AMOEBA). This corresponds to an approximate estimate *via* the Boltzmann equation (where *ρ*_*p*_ and *ρ*_*NP*_ are the water densities for the polarizable and non-polarizable models respectively):

$$\Delta U=-RT\ln\left[\rho_{P}/\rho_{NP}\right]$$

of stabilisation of the nanoconfined water within the hydrophobic gate of ~-1.6 kJ/mol by including polarization. A comparable estimate for TMEM175 yields a stabilisation of ~-4.2 kJ/mol by including polarization. An approximate calculation of the relative potential energies of a water dipole in a single molecule sized cavity in dielectrics of 1 (*i.e. in vacuo) vs*. 4 (for a hydrophobic cavity in a protein) yields about -7 kJ/mol stabilisation of the latter case.^60^ Thus, the same trend is seen for the two nanopore systems but the stabilisation by including polarization is more marked in the extreme nanoconfinement case of the closed hydrophobic gate of TMEM175, which can accommodate ~1-4 water molecules compared with the (partially) open hydrophobic gate in the 5HT3R 6DG8 structure which can more readily accommodate multiple water molecules.

Comparisons with experimental data remain difficult but we note that measurements of the refractive index of nanoconfined water (corresponding to its dielectric constant at optical frequencies^47^) suggest a decrease in molecular polarizability corresponding to a reduction in refractive index from *n* = 1.33 (bulk) to *n* = ~1.25 for water confined in ~10 nm gaps of hydrophobic interfaces. It would therefore be of great interest to know how the refractive index of water behaves on nanoconfinement scales of < 1 nm.

### Trajectories of De-wetting

We examined the de-wetting behaviour of trapped waters in the hydrophobic nanocavity using the polarizable AMOEBA14 model in a little more detail (Fig. 7 and SI Fig. S11). We tracked the trajectories projected onto the *z* axis of all water molecules initially in the hydrophobic gate region. In two of the three simulations, 3-4 waters were initially present but 1-2 of these exited within the first ~40 ns. In the third repeat the hydrophobic gate was fully de-wetted at the start and remained so for the duration (50 ns) of the simulation (SI Fig. S11).

We also examined the first repeat of these simulations in more detail (Fig. 7). At the start of the simulation three water molecules were present within the central hydrophobic nanocavity. This is divided into two ‘sub-cavities’, one formed by the I and L sidechain rings and one by the L and L sidechain rings. Just before 40 ns, one water molecule exits from the hydrophobic gate *via* the wider L side. The remaining two water molecules then continue to switch between the two sub-cavities: sometimes one in each (*e*.*g*. Fig. 7D), sometimes both water molecules in one sub-cavity (*e*.*g*. Fig. 7E), with the I-L sub-cavity being much more commonly occupied. In the latter case the two waters form a hydrogen bond. Similar behaviour was seen in the second repeat simulation (SI Fig. S11B), during which two water molecules leave early on (< 10 ns) and then two remaining waters switch back and forth dynamically between the same or different nanocavities.

By contrast, in the TIP4P simulations (SI Fig. S1A), in 5 (out of 6) simulations trapped waters were present but were expelled in <15 ns in each case. In the TIP4P/2005 simulations (SI Fig. S1B), in 4 (out of 6) simulations initial trapped water molecules were expelled, whereas in one repeat they mostly remained trapped for the entire simulation. Taken together, these results are consistent with the overall instability of the wetted state of the hydrophobic nanocavity. A metastable state with one or more trapped waters may exist, the exact stability of which is sensitive to the water model employed.

## Conclusions

From the results described above, it can be seen that the broad picture of hydrophobic gating (explored for a closed state of a hydrophobic gate in an ion channel protein) is robust to changes in additive (rigid fixed-charge) water models. However, quantitative details alter when electronic polarizability is included in the simulations. This in turn suggests the need to include consideration of polarizability (see *e*.*g*. ^61^ for a recent example) when designing hydrophobic gates or comparable structures into nanopores.^14, 62^ Relatively small changes in detailed nanoscale structure (and hence in the local dielectric/polarizability properties) could be used to ‘fine tune’ a hydrophobic gate to *e*.*g*. electrowetting.

It would be interesting to consider how an applied electric field or pressure would affect differences in water model on hydrophobic gating in TMEM175. Under physiological conditions, with a membrane potential of ~50-200 mV, this would equate to applied electric fields of ~0.005-0.02 V/nm or pressures of ~2-9 MPa. In a previous study on the 5-HT_3_ receptor channel, the effect of supraphysiological electric fields was investigated using different rigid fixed-charge water models;^24^ this implied that different water models did affect the wetting/de-wetting behaviour. Under high pressures of 110-220 MPa, it has been found that water flow rates through a carbon nanotube significantly depend on the rigid fixed-charge water model employed.^63–64^ However, to our knowledge, there have not yet been any studies into the effect of water models on hydrophobic gating in ion channels under physiologically relevant electric fields or pressures. Based on our previous experience, it is possible that under an applied electric field or pressure, the choice of water model could have an effect on hydrophobic gating, particularly if additive and polarizable water models were compared. This would be of importance to the development of designing electrowetting pores, particularly in the case of synthetic pores.

In this study we have used an X-ray structure of a prokaryotic TMEM175 structure (CmTMEM175 at 3.3 Å resolution; PBD 5VRE^40^) as a model for investigating the effect of mutating the pore lining and water model on wetting/de-wetting behaviour. CryoEM studies of the human TMEM175 channel^41^ reveal two possible open and closed structures. Further TMEM175 structures (MtTMEM175) have been solved by X-ray diffraction to 2.4 Å resolution (PDB 6HD8^42^). These differ from the 5VRE CmTMEM175 structure in some respects. In particular, the pore-lining isoleucine (Ile23) in CmTMEM175 is replaced by a leucine (Leu35) in MtTMEM175. Together these structures indicate conservation of a hydrophobic nanoscale constriction in the transmembrane pore.

The current studies could be extended in the future by applying polarizable (AMOEBA) simulations to characterize water behaviour in a range of channel structures selected to have hydrophobic gates of differing dimensions.^22^ This could enable us to explore the effect of the *degree* of nanoconfinement on the extent of anomalous water behaviour. Of course, one must also reflect on whether polarizable models are better able to replicate experimental data than additive models. The AMOEBA14 water model is able to accurately reproduce experimental bulk water properties across a range of temperatures.^45^ In the context of simulations involving several chemical entities, we note that *e*.*g*. local accumulation of anions at water/graphene interfaces as demonstrated experimentally^65^ requires a simple model of polarisation (namely charge scaling^66^) to be reproduced in simulations. Furthermore, inclusion of polarizability (using AMOEBA) seems to be necessary to provide a consistent and accurate explanation of ion permeation through potassium channels.^67^

Combining recent computational (this study) and experimental (*e*.*g*. ^58^) approaches would therefore allow us to exploit biological nanopores/channels to characterise the effects of confinement on water behaviour on smaller scales than those currently addressed (down to ~1.5 nm) by studies of nanofabricated devices.^46^ In the limiting case, this may enable us to use biological systems to examine the behaviour of water under extreme nanoconfinement with just one or two water molecules present. This is of relevance to a number of areas of enquiry, especially given the continued interest in the behaviour of water confined in nanoporous environments.^68–69^ Furthermore, in a recent experimental study^70^ it has been suggested that nanocavities may be present within other membrane proteins (*e*.*g*. GPCRs) and may contribute to the activation mechanism of these receptors. Thus, it is important to understand membrane protein nanocavities both from the perspective of nanopore design, and also for understanding mechanisms of protein nanoswitches within biological membranes.

## Methods

### Protein structure preparation

The prokaryotic TMEM175 structure from *Chamaesiphon minutus* (CmTMEM175 PBD: 5VRE) was run through the WHATIF server (https://swift.cmbi.umcn.nl/whatif/) to fix any missing sidechains.^71^ The three rings of pore-lining residues forming the hydrophobic gate in the wild-type (Ile23, Leu 27 and Leu30 on each of the four subunits) were then mutated in PyMol (http://www.pymol.org) to three rings of alanines, asparagines or valines to form the three mutant structures (‘AAA’, ‘NNN’ and ‘VVV’ structures).

### Non-polarizable simulations

TMEM175 structures were embedded in a POPC (1-palmitoyl-2-oleoyl-*sn*-glycero-3-phosphocholine) bilayer using the MemProtMD and CG2AT protocols.^72–73^ This uses a multi-scale methodology to solvate the system with ~0.15 M NaCl, self-assemble the lipid bilayer and equilibrate the system. Production runs involving the additive water models were performed using GROMACS^74^ version 2016.3 with the OPLS all-atom protein force field with united-atom lipids.^75^ We chose to use the OPLS force field because this was developed to accurately reproduce experimental water properties within biological systems. Each simulation was run for 100 ns with an integration time step of 2 fs. For the CHAP analysis (see below), the first 10 ns were discarded as equilibration. 6 repeats were run for each protein structure and water model combination, resulting in a total of 120 simulations. The temperature was maintained at 310 K using the V-rescale thermostat^76^ and a time coupling constant of 0.1 ps. The pressure was maintained at 1 bar using the semi-isotropic Parrinello-Rahman barostat^77^ and a time coupling constant of 1 ps. The Verlet cut-off scheme was used, along with the Particle Mesh Ewald method for electrostatics.^78^ The LINCS algorithm was used to constrain bonds.^79^

The positions of the protein backbone atoms were position restrained with a force constant of 1000 kJ mol^-1^ nm^-2^, however protein side chains were allowed to relax. We chose to apply these position restraints because we did not want our overall simulation protein structure to deviate from the original experimental coordinates. It was important to do this because we specifically wanted to assess how changes in the protein pore lining (*i.e. via* mutants) and water model affected the wetting/de-wetting behaviour within a given overall protein geometry.

### Polarizable simulations

These were performed using a similar method to that described in Klesse *et al*.^30^ (https://github.com/Inniag/openmm-scripts-amoeba). The wild-type and VVV mutant structures were embedded in a DOPC bilayer and solvated with ~0.15 M NaCl solution using the bilayer self-assembly method outlined for the non-polarizable simulations. A 10 ns equilibration simulation was then run using GROMACS with the non-polarizable CHARMM36m force field. Polarizable production runs were performed using OpenMM,^31^ and lasted for 50 ns for the wild-type structure and 35-50 ns for the VVV mutant structure. The AMOEBA force field was used for all atoms in the simulation *i.e*. for the protein,^80^ ions^81^ and lipids,^82^ together with the AMOEBA14 water model.^45^ The r-RESPA algorithm was used with an outer timestep of 2 fs and an inner timestep of 0.25 fs. The pressure was maintained at 1 bar using the Monte Carlo barostat and the temperature was maintained at 310 K using the Andersen thermostat. The Cα backbone atoms were position restrained with a force constant of 1000 kJ mol^-1^ nm^-2^ for both the equilibration and production runs. There were 3 repeats for each structure, resulting in a total of 6 simulations.

### Analysis

Profiles for pore radius, water density and free energy were obtained using ChAP (www.channotation.org).^27^ In all cases, the first 10 ns were discarded from the analysis and treated as equilibration. A bandwidth of 0.14 nm was used for calculating the water density through the pore. The free energy profiles were obtained *via*:

$$G(s)=-k_{B}T\ln[n(s)]+k_{B}T\ln[C]$$

where *G*(*s*) is the free energy of wetting/de-wetting, *n*(*s*) is the water density, *s* is the coordinate passing down the central axis of the pore (approximately perpendicular to the bilayer), *T* is the temperature, *k_B_* is the Boltzmann constant and *C* is an arbitrary constant set so that *G*(*s*) = 0 outside the pore. Having first discarded the first 10 ns of the simulation as equilibration, these profiles were averaged over all remaining frames of the simulation, and then averaged over all repeats as appropriate.

The positions of individual water molecules were calculated with the aid of MDAnalysis.^83–85^ It should be noted that the protein is oriented such that *z* axis is approximately antiparallel to the local pore *s* defined by CHAP and so for all practical purposes *z = -s*. Figures were created using VMD^86^ and Matplotlib^87^ (10.5281/zenodo.592536).

## Supplementary Material

The Supporting Information is available free of charge at https://pubs.acs.org. It includes SI Figures S1 to S11. S1: additional water trajectories for the wild-type channel using TIP4P and TIP4P/2005; S2: radius, water density and free energy profiles for the wild-type channel as a function of water model; S3-4: water density and free energy profiles for the wild-type channel as a function of water model including standard deviations; S5-10: radius, water density and free energy profiles as a function of water model, including standard deviations for the free energy, for the AAA mutant (S5-6), NNN mutant (S7-8) and VVV mutant (S9-10); S11: additional water trajectories for the wild-type channel using AMOEBA.

SI

## References

[1] Nazari M, Davoodabadi A, Huang DZ, Luo TF, Ghasemi H (2020). Transport Phenomena in Nano/Molecular Confinements. ACS Nano.

[2] Hou X, Guo W, Jiang L (2011). Biomimetic Smart Nanopores and Nanochannels. Chem Soc Rev.

[3] Pohl P (2004). Combined Transport of Water and Ions through Membrane Channels. Biol Chem.

[4] Rasaiah JC, Garde S, Hummer G (2008). Water in Nonpolar Confinement: From Nanotubes to Proteins and Beyond. Annu Rev Phys Chem.

[5] Faucher S, Aluru N, Bazant MZ, Blankschtein D, Brozena AH, Cumings J, de Souza JP, Elimelech M, Epsztein R, Fourkas JT, Rajan AG (2019). Critical Knowledge Gaps in Mass Transport through Single-Digit Nanopores: A Review and Perspective. J Phys Chem C.

[6] Lynch C, Rao S, Sansom MSP (2020). Water in Biological Channels and Nanopores: A Molecular Simulation Perspective. Chem Rev.

[7] Olivieri J-F, Hynes JT, Laage D (2021). Confined Water’s Dielectric Constant Reduction Is Due to the Surrounding Low Dielectric Media and Not to Interfacial Molecular Ordering. J Phys Chem Lett.

[8] Aryal P, Sansom MSP, Tucker SJ (2014). Hydrophobic Gating in Ion Channels. J Molec Biol.

[9] Trick JL, Chelvaniththilan S, Klesse G, Aryal P, Wallace EJ, Tucker SJ, Sansom MSP (2016). Functional Annotation of Ion Channel Structures by Molecular Simulation. Structure.

[10] Zhu FQ, Hummer G (2012). Drying Transition in the Hydrophobic Gate of the GLIC Channel Blocks Ion Conduction. Biophys J.

[11] Powell MR, Cleary L, Davenport M, Shea KJ, Siwy ZS (2011). Electric-Field-Induced Wetting and Dewetting in Single Hydrophobic Nanopores. Nature Nanotech.

[12] Rao SL, Klesse G, Lynch CI, Tucker SJ, Sansom MSP (2021). Molecular Simulations of Hydrophobic Gating of Pentameric Ligand Gated Ion Channels: Insights into Water and Ions. J Phys Chem B.

[13] Trick JL, Wallace EJ, Bayley H, Sansom MSP (2014). Designing a Hydrophobic Barrier within Biomimetic Nanopores. ACS Nano.

[14] Xu C, Lu P, Gamal El-Din TM, Pei XY, Johnson MC, Uyeda A, Bick MJ, Xu Q, Jiang D, Bai H, Reggiano G (2020). Computational Design of Transmembrane Pores. Nature.

[15] Vorobieva AA, White P, Liang BY, Horne JE, Bera AK, Chow CM, Gerben S, Marx S, Kang A, Stiving AQ, Harvey SR (2021). De Novo Design of Transmembrane Beta Barrels. Science.

[16] Beckstein O, Biggin PC, Sansom MSP (2001). A Hydrophobic Gating Mechanism for Nanopores. J Phys Chem B.

[17] Beckstein O, Sansom MSP (2003). Liquid–Vapor Oscillations of Water in Hydrophobic Nanopores. Proc Nat Acad Sci USA.

[18] Beckstein O, Sansom MSP (2004). The Influence of Geometry, Surface Character and Flexibility on the Permeation of Ions and Water through Biological Pores. Phys Biol.

[19] Allen R, Melchionna S, Hansen JP (2002). Intermittent Permeation of Cylindrical Nanopores by Water. Phys Rev Lett.

[20] Allen R, Hansen JP, Melchionna S (2003). Molecular Dynamics Investigation of Water Permeation through Nanopores. J Chem Phys.

[21] Corry B (2006). An Energy-Efficient Gating Mechanism in the Acetylcholine Receptor Channel Suggested by Molecular and Brownian Dynamics. Biophys J.

[22] Rao S, Klesse G, Stansfeld PJ, Tucker SJ, Sansom MSP (2019). A Heuristic Derived from Analysis of the Ion Channel Structural Proteome Permits the Rapid Identification of Hydrophobic Gates. Proc Natl Acad Sci USA.

[23] Trick JL, Song C, Wallace EJ, Sansom MSP (2017). Voltage Gating of a Biomimetic Nanopore: Electrowetting of a Hydrophobic Barrier. ACS Nano.

[24] Klesse G, Tucker SJ, Sansom MSP (2020). Electric Field Induced Wetting of a Hydrophobic Gate in a Model Nanopore Based on the 5-HT3 Receptor Channel. ACS Nano.

[25] Petrov E, Rohde PR, Martinac B (2011). Flying-Patch Patch-Clamp Study of G22E-MscL Mutant under High Hydrostatic Pressure. Biophys J.

[26] Macdonald A (2021). Effects of High Pressure on the Activity of Ordinary Animals, Including Humans, and on the Function of Their Excitable Cells and Ion Channels. Life at High Pressure.

[27] Klesse G, Rao S, Sansom MSP, Tucker SJ (2019). CHAP: A Versatile Tool for the Structural and Functional Annotation of Ion Channel Pores. J Molec Biol.

[28] Rao S, Lynch CI, Klesse G, Oakley GE, Stansfeld PJ, Tucker SJ, Sansom MSP (2018). Water and Hydrophobic Gates in Ion Channels and Nanopores. Faraday Disc.

[29] Calvelo M, Lynch CI, Granja JR, Sansom MSP, Garcia-Fandiño R (2021). Effect of Water Models on Transmembrane Self-Assembled Cyclic Peptide Nanotubes. ACS Nano.

[30] Klesse G, Rao S, Tucker SJ, Sansom MSP (2020). Induced Polarization in Molecular Dynamics Simulations of the 5-HT_3_ Receptor Channel. J Amer Chem Soc.

[31] Eastman P, Swails J, Chodera JD, McGibbon RT, Zhao YT, Beauchamp KA, Wang LP, Simmonett AC, Harrigan MP, Stern CD, Wiewiora RP (2017). OpenMM 7: Rapid Development of High Performance Algorithms for Molecular Dynamics. PLoS Comput Biol.

[32] Adjoua O, Lagardère L, Jolly L-H, Durocher A, Very T, Dupays I, Wang Z, Inizan TJ, Célerse F, Ren P, Ponder JW (2021). Accelerating Molecular Dynamics Simulations of Large Complex Systems with Advanced Point Dipole Polarizable Force Fields Using GPUs and Multi-GPU Systems. J Chem Theor Comput.

[33] Chen PR, Vorobyov I, Roux B, Allen TW (2021). Molecular Dynamics Simulations Based on Polarizable Models Show That Ion Permeation Interconverts between Different Mechanisms as a Function of Membrane Thickness. J Phys Chem B.

[34] Ngo V, Li H, MacKerell AD, Allen TW, Roux B, Noskov S (2021). Polarization Effects in Water-Mediated Selective Cation Transport across a Narrow Transmembrane Channel. J Chem Theor Comput.

[35] Bradshaw RT, Dziedzic J, Skylaris CK, Essex JW (2020). The Role of Electrostatics in Enzymes: Do Biomolecular Force Fields Reflect Protein Electric Fields?. J Chem Inform Model.

[36] Daub CD, Cann NM, Bratko D, Luzar A (2018). Electrokinetic Flow of an Aqueous Electrolyte in Amorphous Silica Nanotubes. Phys Chem Chem Phys.

[37] Moucka F, Zamfir S, Bratko D, Luzar A (2019). Molecular Polarizability in Open Ensemble Simulations of Aqueous Nanoconfinements under Electric Field. J Chem Phys.

[38] Ailenei A-E, Beu TA (2021). Ion Transport through Gated Carbon Nanotubes: Molecular Dynamics Simulations Using Polarizable Water. J Mol Struct.

[39] Çinaroğlu SS, Biggin PC (2021). Evaluating the Performance of Water Models with Host–Guest Force Fields in Binding Enthalpy Calculations for Cucurbit[7]uril–Guest Systems. J Phys Chem B.

[40] Lee C, Guo J, Zeng W, Kim S, She J, Cang C, Ren D, Jiang Y (2017). The Lysosomal Potassium Channel TMEM175 Adopts a Novel Tetrameric Architecture. Nature.

[41] Oh S, Paknejad N, Hite RK (2020). Gating and Selectivity Mechanisms for the Lysosomal K^+^ Channel TMEM175. Elife.

[42] Brunner JD, Jakob RP, Schulze T, Neldner Y, Moroni A, Thiel G, Maier T, Schenck S (2020). Structural Basis for Ion Selectivity in TMEM175 K+ Channels. Elife.

[43] Wie J, Liu Z, Song H, Tropea TF, Yang L, Wang H, Liang Y, Cang C, Aranda K, Lohmann J, Yang J (2021). A Growth-Factor-Activated Lysosomal K^+^ Channel Regulates Parkinson’s Pathology. Nature.

[44] Chipman DM (2013). Water from Ambient to Supercritical Conditions with the AMOEBA Model. Journal of Physical Chemistry B.

[45] Laury ML, Wang LP, Pande VS, Head-Gordon T, Ponder JW (2015). Revised Parameters for the AMOEBA Polarizable Atomic Multipole Water Model. J Phys Chem B.

[46] Fumagalli L, Esfandiar A, Fabregas R, Hu S, Ares P, Janardanan A, Yang Q, Radha B, Taniguchi T, Watanabe K, Gomila G (2018). Anomalously Low Dielectric Constant of Confined Water. Science.

[47] Le THH, Morita A, Tanaka T (2020). Refractive Index of Nanoconfined Water Reveals its Anomalous Physical Properties. Nanoscale Horizons.

[48] Ghasemi S, Alihosseini M, Peymanirad F, Jalali H, Ketabi SA, Khoeini F, Neek-Amal M (2020). Electronic, Dielectric, and Optical Properties of Two-Dimensional and Bulk Ice: A Multiscale Simulation Study. Phy Rev B.

[49] Gonzalez MA, Abascal JLF (2011). A Flexible Model for Water Based on TIP4P/2005. J Chem Phys.

[50] Hu HY, Wang F (2015). The Liquid-Vapor Equilibria of TIP4P/2005 and BLYPSP-4F Water Models Determined through Direct Simulations of the Liquid-Vapor Interface. J Chem Phys.

[51] Zaragoza A, Gonzalez MA, Joly L, Lopez-Montero I, Canales MA, Benavides AL, Valeriani C (2019). Molecular Dynamics Study of Nanoconfined TIP4P/2005 Water: How Confinement and Temperature Affect Diffusion and Viscosity. Phys Chem Chem Phys.

[52] Izadi S, Anandakrishnan R, Onufriev AV (2014). Building Water Models: A Different Approach. J Phys Chem Lett.

[53] Jorgensen WL, Chandresekhar J, Madura JD, Impey RW, Klein ML (1983). Comparison of Simple Potential Functions for Simulating Liquid Water. J Chem Phys.

[54] Berendsen HJC, Grigera JR, Straatsma TP (1987). The Missing Term in Effective Pair Potentials. J Phys Chem.

[55] van der Spoel D, van Maaren PJ, Berendsen HJC (1998). A Systematic Study of Water Models for Molecular Simulation: Derivation of Water Models Optimized for Use with a Reaction Field. J Chem Phys.

[56] Izadi S, Onufriev AV (2016). Accuracy Limit of Rigid 3-Point Water Models. J Chem Phys.

[57] Onufriev AV, Izadi S (2018). Water Models for Biomolecular Simulations. WIREs Comput Molec Sci.

[58] Zhang XN, Zhang Y, Tang SY, Ma SJ, Shen Y, Chen YK, Tong Q, Li YZ, Yang J (2021). Hydrophobic Gate of Mechanosensitive Channel of Large Conductance in Lipid Bilayers Revealed by Solid-State NMR Spectroscopy. J Phys Chem B.

[59] He LL, Li Y, Zhao DX, Yu L, Zhao CL, Lu LN, Liu C, Yang ZZ (2019). Structure and Phase Behavior of the Confined Water in Graphene Nanocapillaries Studied by ABEEMσπ Polarizable Force Field. J Phys Chem C.

[60] Ernst JA, Clubb RT, Zhou HX, Gronenborn AM, Clore GM (1995). Demonstration of Positionally Disordered Water within a Protein Hydrophobic Cavity by NMR. Science.

[61] Polster JW, Acar ET, Aydin F, Zhan C, Pham TA, Siwy ZS (2020). Gating of Hydrophobic Nanopores with Large Anions. ACS Nano.

[62] Scott AJ, Niitsu A, Kratochvil HT, Lang EJM, Sengel JT, Dawson WM, Mahendran KR, Mravic M, Thomson AR, Brady RL, Liu L (2021). Constructing Ion Channels from Water-Soluble α-Helical Barrels. Nature Chem.

[63] Liu L, Patey GN (2016). Simulated Conduction Rates of Water through a (6,6) Carbon Nanotube Strongly Depend on Bulk Properties of the Model Employed. J Chem Phys.

[64] Liu L, Patey GN (2017). A Molecular Dynamics Investigation of the Influence of Water Structure on Ion Conduction through a Carbon Nanotube. J Chem Phys.

[65] McCaffrey DL, Nguyen SC, Cox SJ, Weller H, Alivisatos AP, Geissler PL, Saykally RJ (2017). Mechanism of Ion Adsorption to Aqueous Interfaces: Graphene/Water vs. Air/Water. Proc Natl Acad Sci USA.

[66] Kirby BJ, Jungwirth P (2019). Charge Scaling Manifesto: A Way of Reconciling the Inherently Macroscopic and Microscopic Natures of Molecular Simulations. J Phys Chem Lett.

[67] Jing Z, Rackers JA, Pratt LR, Liu C, Rempe SB, Ren P (2021). Thermodynamics of Ion Binding and Occupancy in Potassium Channels. Chem Sci.

[68] Faucher S, Kuehne M, Koman VB, Northrup N, Kozawa D, Yuan Z, Li SX, Zeng YW, Ichihara T, Misra RP, Aluru N (2021). Diameter Dependence of Water Filling in Lithographically Segmented Isolated Carbon Nanotubes. ACS Nano.

[69] Coudert F-X, Boutin A, Fuchs AH (2021). Open Questions on Water Confined in Nanoporous Materials. Comms Chemistry.

[70] Abiko LA, Teixeira RD, Engilberge S, Grahl A, Grzesiek S (2021). Filling of a Water-Free Void Explains the Allosteric Regulation of the β1-Adrenergic Receptor by Cholesterol. BioRxiv.

[71] Vriend G (1990). WhatIf - A Molecular Modeling and Drug Design Program. J Mol Graph.

[72] Stansfeld PJ, Sansom MSP (2011). From Coarse-Grained to Atomistic: A Serial Multi-Scale Approach to Membrane Protein Simulations. J Chem Theor Comp.

[73] Stansfeld PJ, Goose JE, Caffrey M, Carpenter EP, Parker JL, Newstead N, Sansom MSP (2015). MemProtMD: Automated Insertion of Membrane Protein Structures into Explicit Lipid Membranes. Structure.

[74] Abraham MJ, Murtola T, Schulz R, Páll S, Smith JC, Hess B, Lindahl E (2015). GROMACS: High Performance Molecular Simulations through Multi-Level Parallelism from Laptops to Supercomputers. SoftwareX.

[75] Jorgensen WL, Maxwell DS, Tirado-Rives J (1996). Development and Testing of the OPLS All-Atom Force Field on Conformational Energetics and Properties of Organic Liquids. J Amer Chem Soc.

[76] Bussi G, Donadio D, Parrinello M (2007). Canonical Sampling through Velocity Rescaling. J Chem Phys.

[77] Parrinello M, Rahman A (1981). Polymorphic Transitions in Single-Crystals - A New Molecular-Dynamics Method. J Appl Phys.

[78] Darden T, York D, Pedersen L (1993). Particle Mesh Ewald - An N.log(N) Method for Ewald Sums in Large Systems. J Chem Phys.

[79] Hess B, Bekker H, Berendsen HJC, Fraaije JGEM (1997). LINCS: A Linear Constraint Solver for Molecular Simulations. J Comp Chem.

[80] Shi Y, Xia Z, Zhang JJ, Best R, Wu CJ, Ponder JW, Ren PY (2013). Polarizable Atomic Multipole-Based AMOEBA Force Field for Proteins. J Chem Theor Comput.

[81] Grossfield A, Ren PY, Ponder JW (2003). Ion Solvation Thermodynamics from Simulation with a Polarizable Force Field. J Amer Chem Soc.

[82] Chu HY, Peng XD, Li Y, Zhang YB, Min HY, Li GH (2018). Polarizable Atomic Multipole-Based Force Field for DOPC and POPE Membrane Lipids. Molec Phys.

[83] Michaud-Agrawal N, Denning EJ, Woolf TB, Beckstein O (2011). MDAnalysis: A Toolkit for the Analysis of Molecular Dynamics Simulations. J Comput Chem.

[84] Gowers RJ, Linke M, Barnoud J, Reddy TJE, Melo MN, Seyler SL, Dotson DL, Domanski J, Buchoux S, Kenney IM, Beckstein O, Austin TX, Benthall S, Rostrup S (2016). MDAnalysis: A Python Package for the Rapid Analysis of Molecular Dynamics Simulations.

[85] Harris CR, Millman KJ, van der Walt SJ, Gommers R, Virtanen P, Cournapeau D, Wieser E, Taylor J, Berg S, Smith NJ, Kern R (2020). Array Programming with NumPy. Nature.

[86] Humphrey W, Dalke A, Schulten K (1996). VMD - Visual Molecular Dynamics. J Molec Graph.

[87] Hunter JD (2007). Matplotlib: A 2D Graphics Environment. Comput Sci Eng.

