# Supplementary material for: Water Nanoconfined in a Hydrophobic Pore: Molecular Dynamics Simulations of Transmembrane Protein 175 and the Influence of Water Models
Source: ACS Nano. Author manuscript; Available in PMC 2021 Dec 30. (PMC7612143; doi:10.1021/acsnano.1c06443)
Supplement: SI [file EMS138803-supplement-SI.pdf]

SUPPORTING INFORMATION

Water Nanoconfined in a Hydrophobic Pore: Molecular Dynamics Simulations of Transmembrane Protein 175 and the Influence of Water Models

Charlotte I. Lynch, Gianni Klesse, Shanlin Rao, Stephen J. Tucker, Mark S. P. Sansom

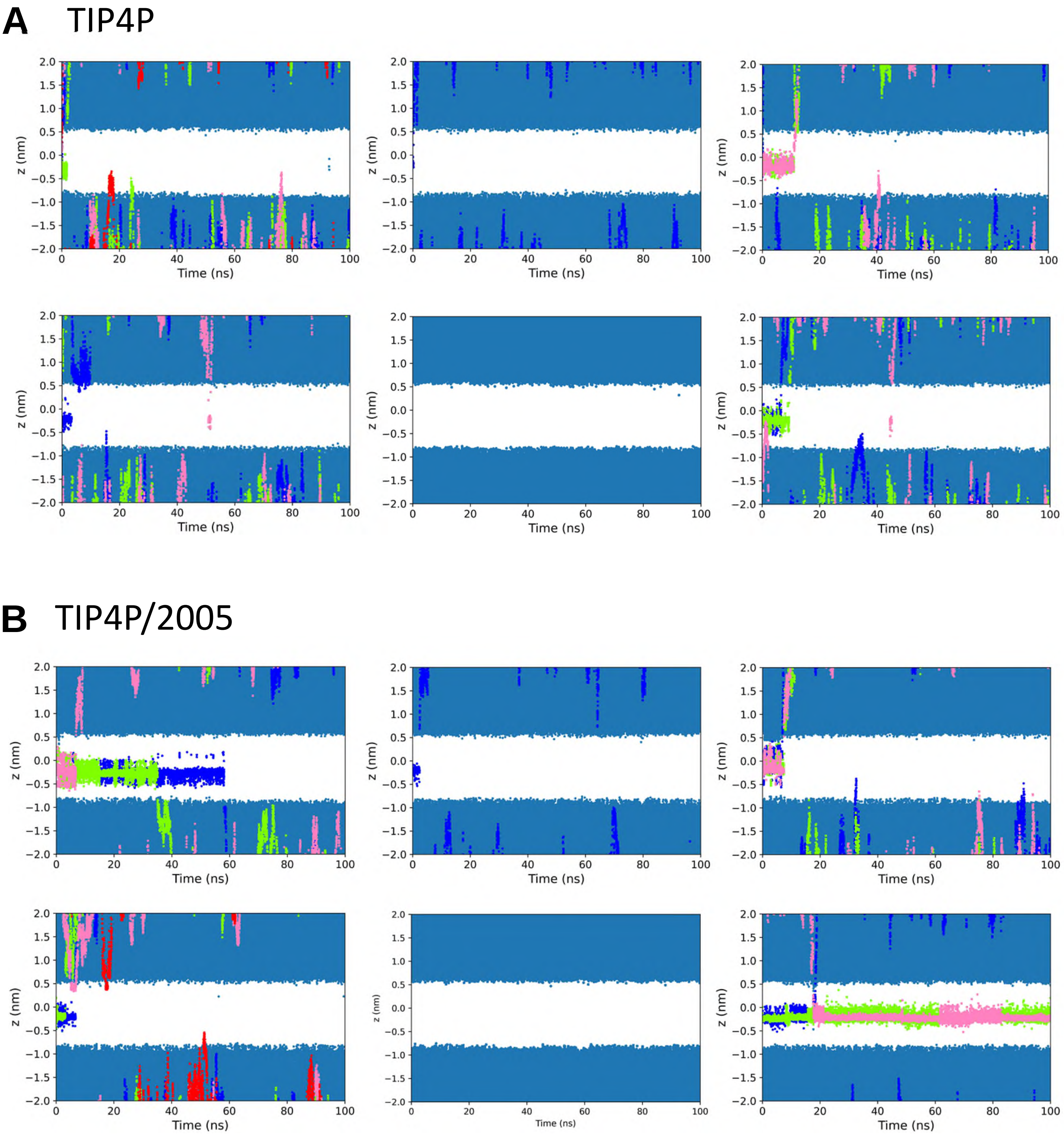

Figure S1:

The trajectories of water molecules in and around the hydrophobic gating region (centred at z = 0) shown projected onto the z axis for each of the 6 repeats of the **A** TIP4P and **B** TIP4P/2005 simulations of the wild-type channel. The trajectories of individual water molecules are highlighted in blue, green, pink and red; the other water molecules are shown in medium blue.

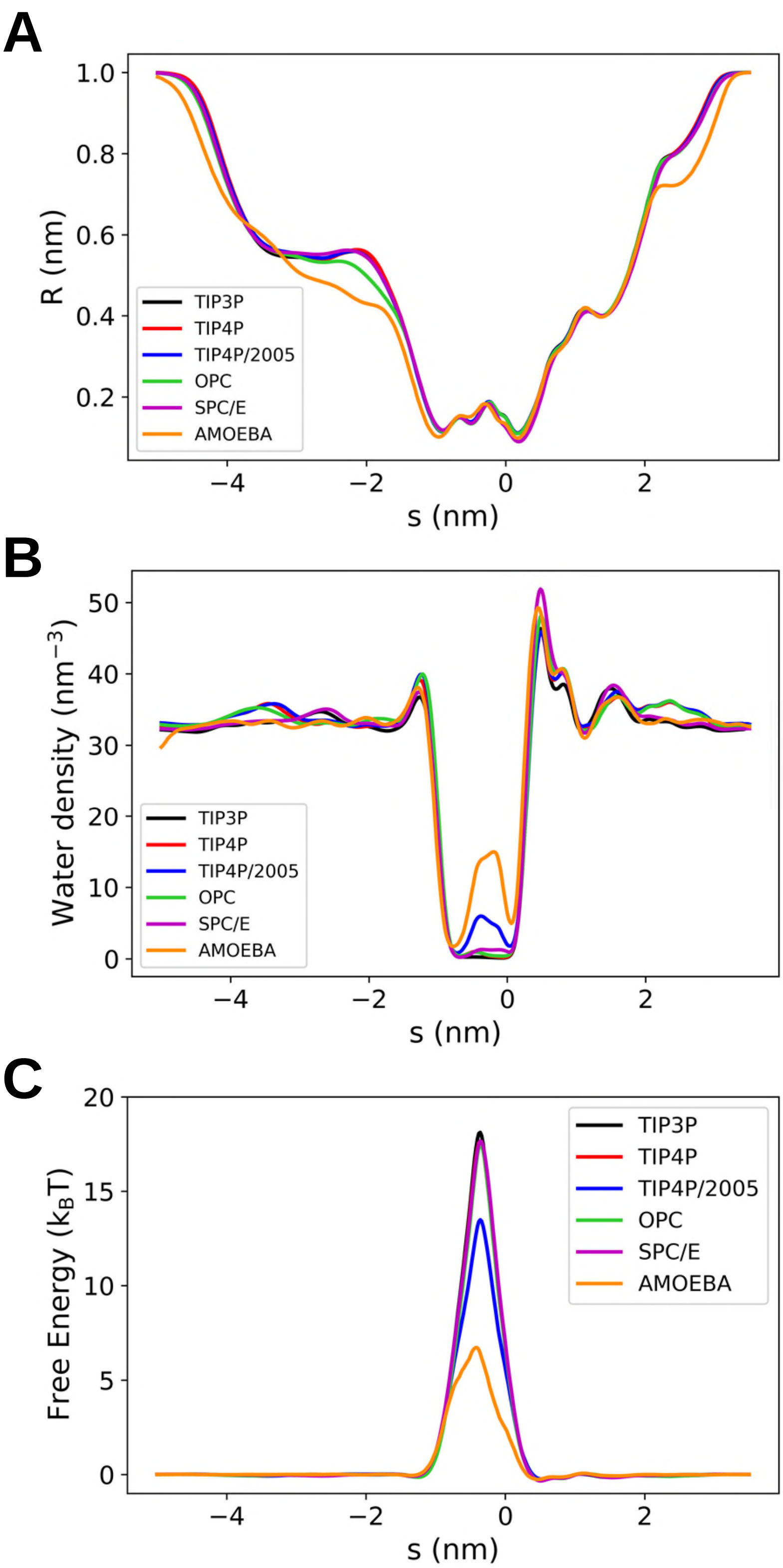

Figure S2:

Profiles for the wild-type channel as a function of water model: **A** radius, **B** water density, **C** water free energy.

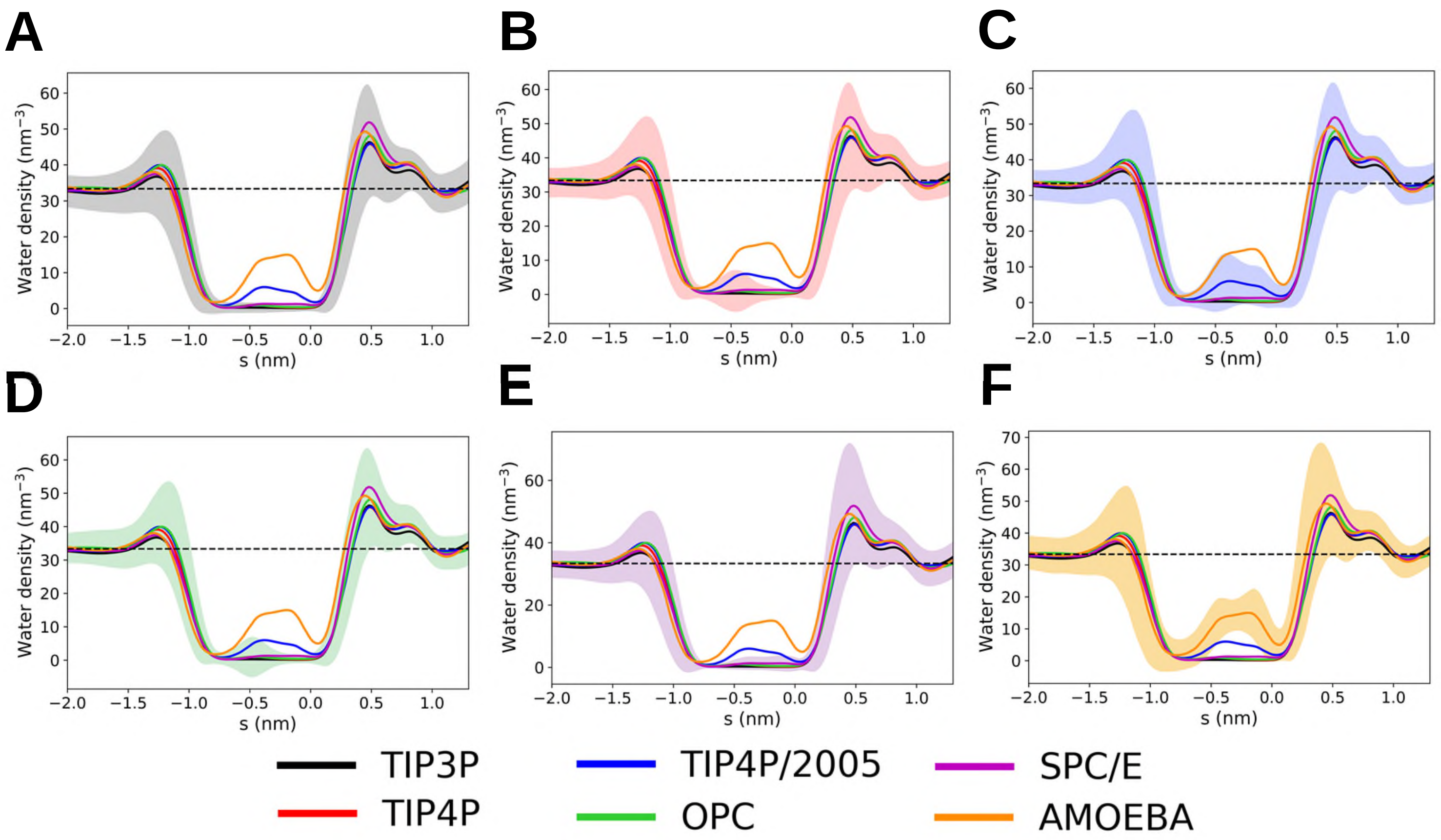

Figure S3:
Water density profiles for the wild-type channel, showing the standard deviation for each water model (shaded regions): **A** TIP3P, **B** TIP4P, **C** TIP4P/2005, **D** OPC, **E** SPC/E and **F** AMOEBA.

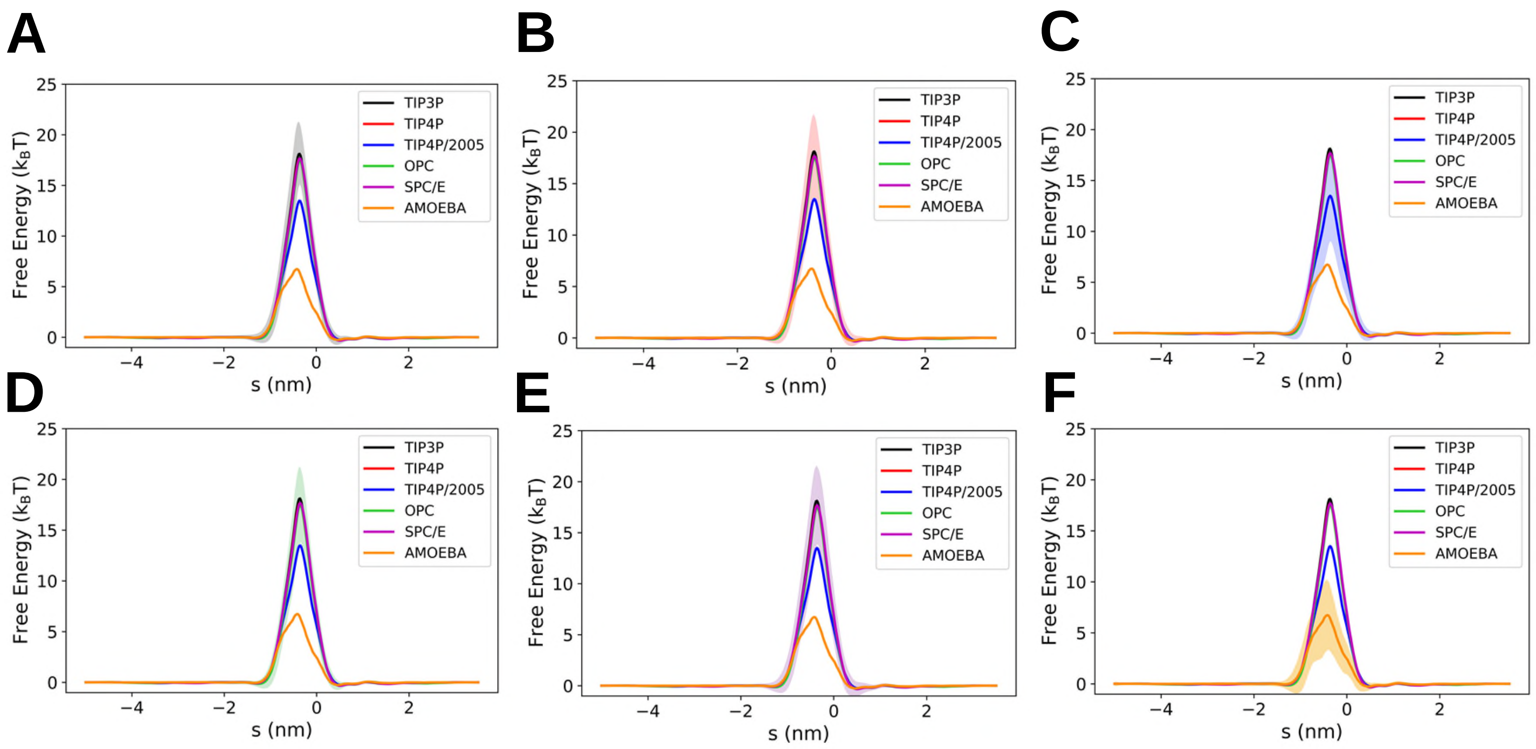

Figure S4:

Water free energy profiles for the wild-type channel, showing the standard deviation for each water model (shaded regions): **A** TIP3P, **B** TIP4P, **C** TIP4P/2005, **D** OPC, **E** SPC/E and **F** AMOEBA.

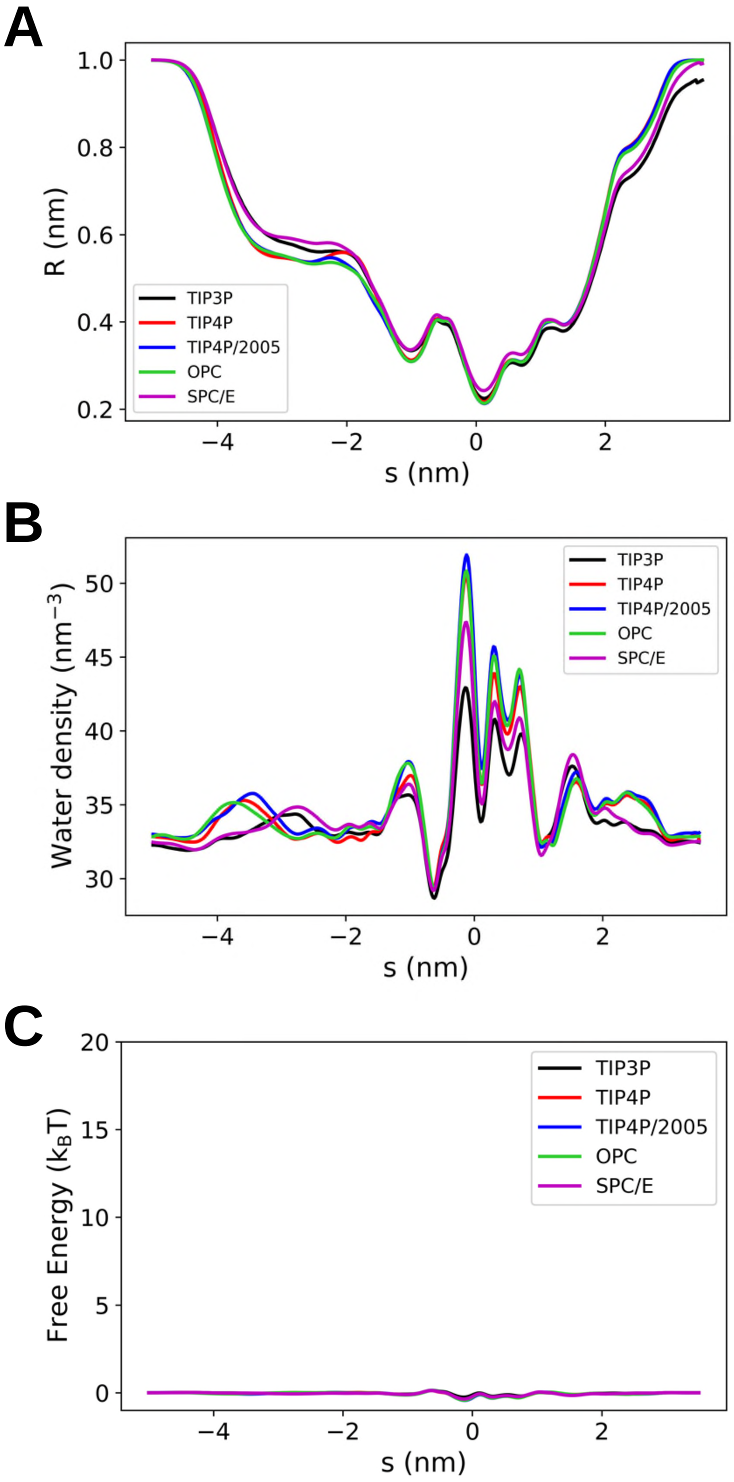

Figure S5:
Profiles for the AAA mutant channel as a function of water model: **A** radius, **B** water density, **C** water free energy.

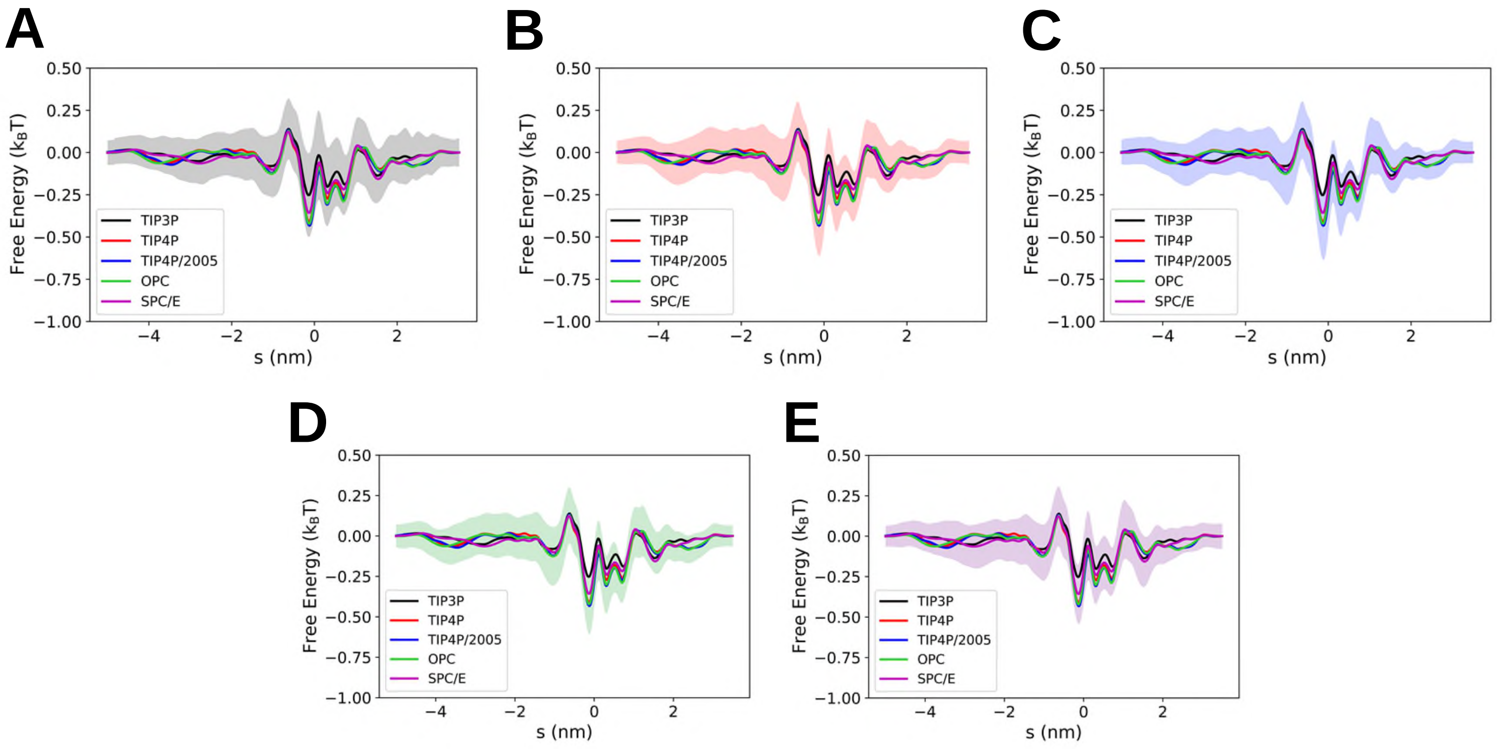

Figure S6:
Water free energy profiles for the AAA mutant channel, showing the standard deviation for each water model (shaded regions): **A** TIP3P, **B** TIP4P, **C** TIP4P/2005, **D** OPC, and **E** SPC/E.

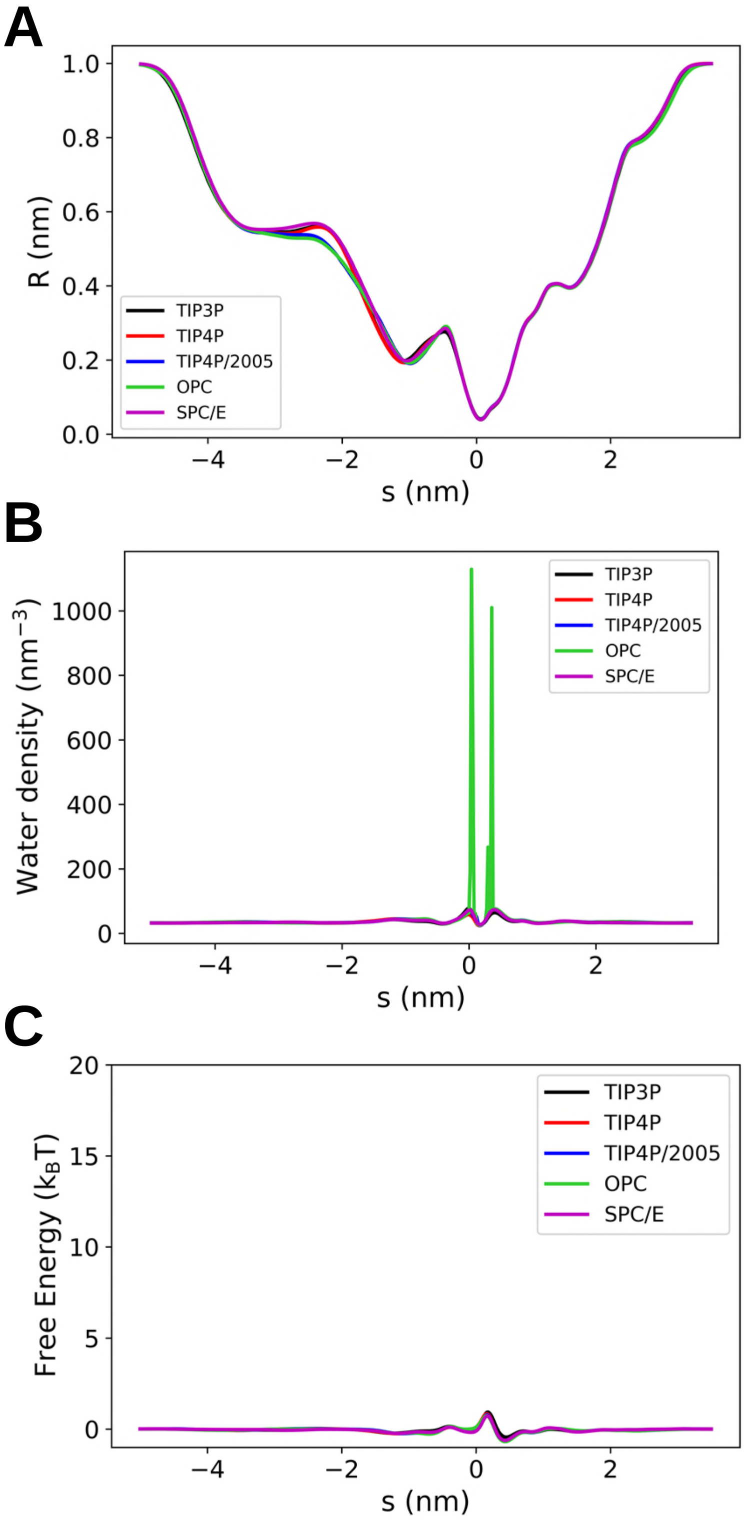

Figure S7:
Profiles for the NNN mutant channel as a function of water model: **A** radius, **B** water density, **C** water free energy.

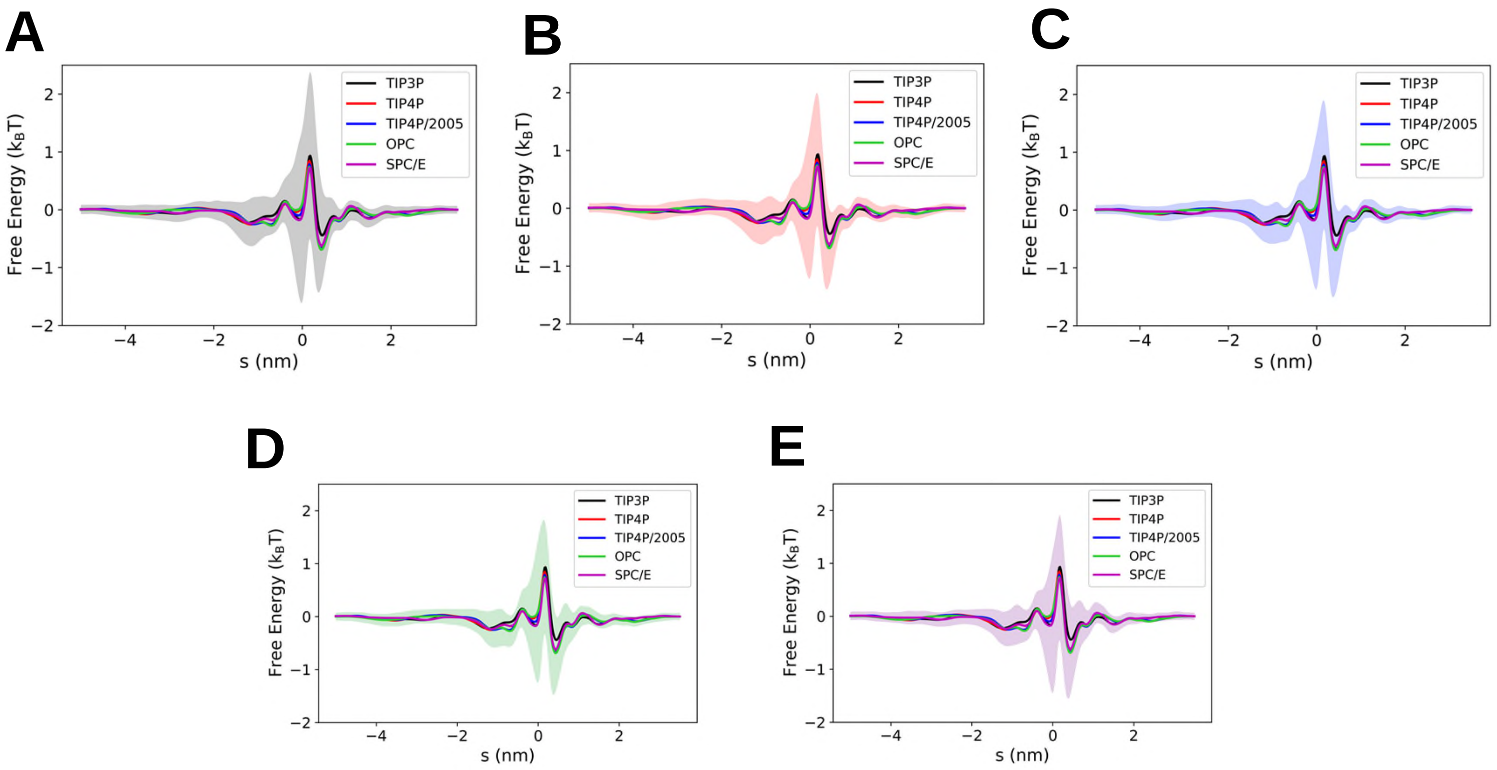

Figure S8:
Water free energy profiles for the NNN mutant channel, showing the standard deviation for each water model (shaded regions): **A** TIP3P, **B** TIP4P, **C** TIP4P/2005, **D** OPC, and **E** SPC/E.

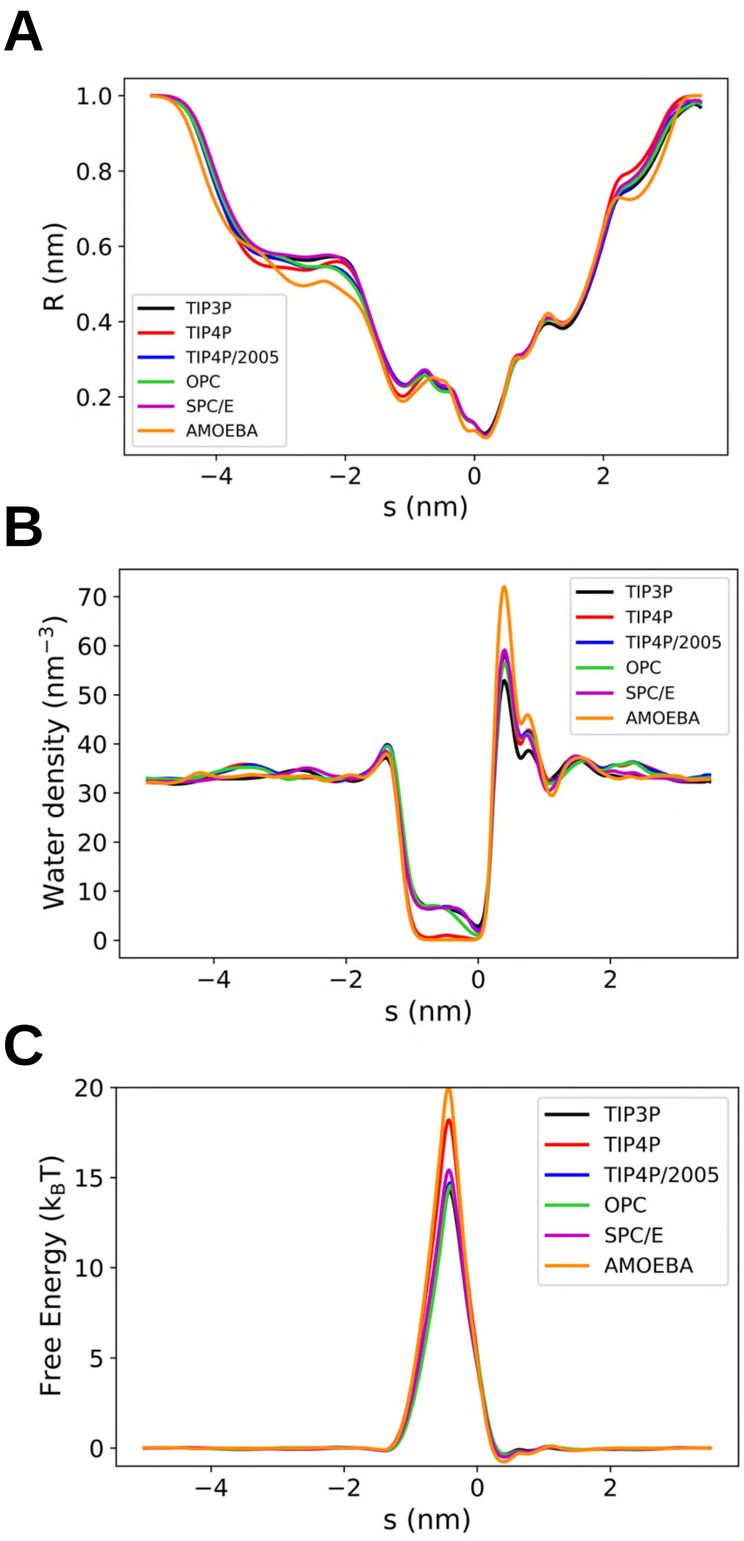

Figure S9:
Profiles for the VVV mutant channel as a function of water model: **A** radius, **B** water density, **C** water free energy.

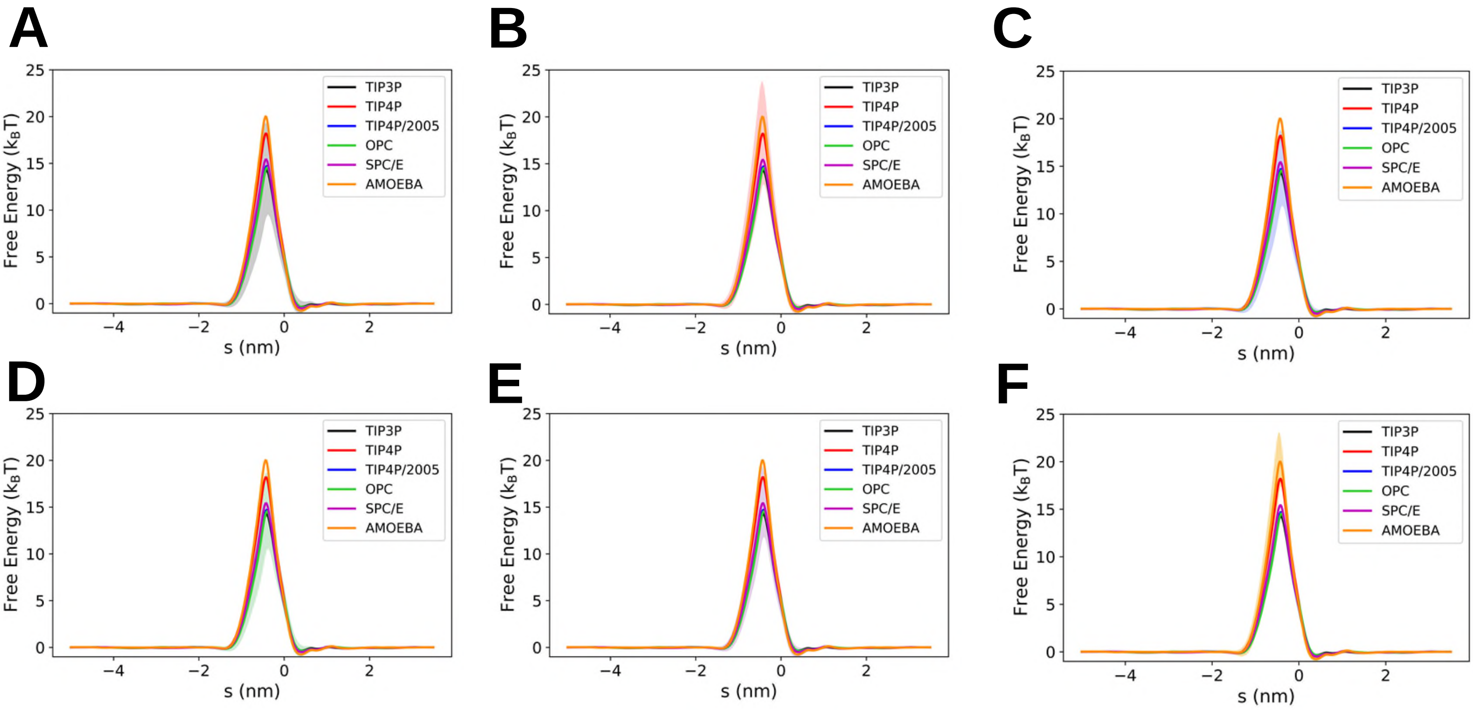

Figure S10:
Water free energy profiles for the VVV mutant channel, showing the standard deviation for each water model (shaded regions): **A** TIP3P, **B** TIP4P, **C** TIP4P/2005, **D** OPC, **E** SPC/E, and **F** AMOEBA.

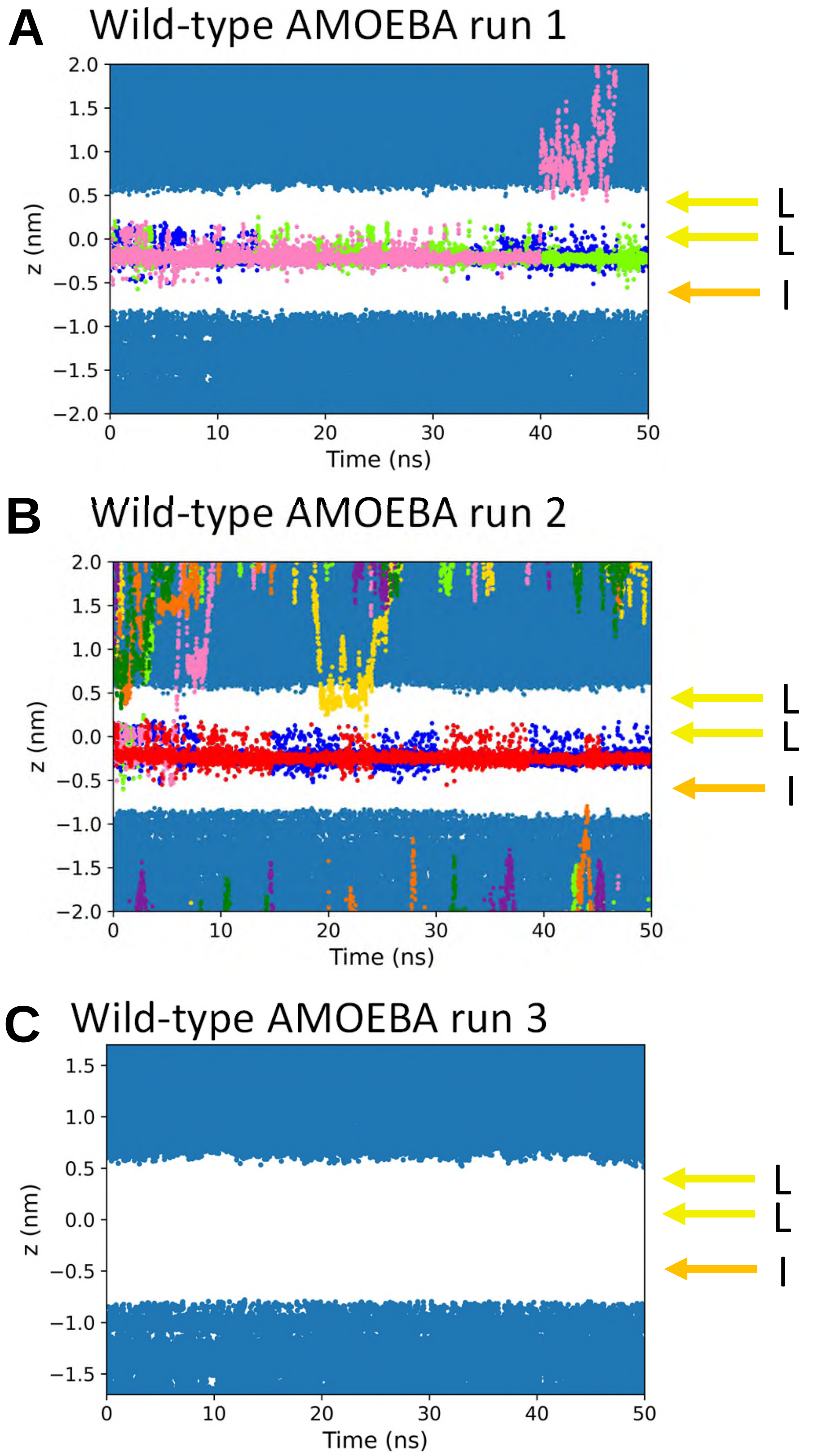

Figure S11:

The trajectories of water molecules initially inside the gating region (bright blue, green, red, pink) are shown projected onto the *z* axis (note that *z* is approximately equivalent to –*s*, the pore axis used in the previous figures) within the region of the hydrophobic gate for each of the 3 repeats of the AMOEBA simulation of the wild-type channel. Water molecules which are situated outside the gating region are shown in dark blue, and those which approach the gating region during the simulation are depicted in other colours. The yellow and orange horizontal arrows show the z-coordinates of the centroid of the hydrophobic L and I rings which form the hydrophobic nanocavity.
